# Supplementary material for: Structural insights into the activation mechanism of antimicrobial GBP1
Source: EMBO J. 2024 Jan 24;43(4):615–36. doi: 10.1038/s44318-023-00023-y (PMC10897159; doi:10.1038/s44318-023-00023-y)
Supplement: Supplementary file 1 — Appendix [file 44318_2023_23_MOESM1_ESM.pdf]

Appendix

Structural insights into the activation mechanism of antimicrobial GBP1

Marius Weismehl[1,2], Xiaofeng Chu[3], Miriam Kutsch[4,5,6,7], Paul Lauterjung[8], Christian Herrmann[8], Misha Kudryashev[3,9], Oliver Daumke[1,2,*]

[1]Structural Biology, Max-Delbrück-Center for Molecular Medicine in the Helmholtz Association (MDC), 13125 Berlin, Germany

[2]Institute for Chemistry and Biochemistry, Freie Universität Berlin, 14195 Berlin, Germany

[3]*In Situ* Structural Biology, Max-Delbrück-Center for Molecular Medicine in the Helmholtz Association (MDC), 13125 Berlin, Germany

[4]Institute of Molecular Pathogenicity, Faculty of Mathematics and Natural Sciences, Heinrich Heine University Düsseldorf, 40225 Düsseldorf, Germany

[5]Institute of Medical Microbiology and Hospital Hygiene, Medical Faculty and University Hospital Düsseldorf, Heinrich Heine University Düsseldorf, 40225 Düsseldorf, Germany

[6]Institute of Biochemistry, Faculty of Mathematics and Natural Sciences, Heinrich Heine University Düsseldorf, 40225 Düsseldorf, Germany

[7]Department of Molecular Genetics and Microbiology, Duke University, 27710 Durham, NC, USA

[8]Faculty of Chemistry and Biochemistry, Physical Chemistry I, Ruhr-University Bochum, 44801 Bochum, Germany

[9]Institute of Medical Physics and Biophysics, Charité-Universitätsmedizin Berlin, 10117 Berlin, Germany

[*] Corresponding Author: oliver.daumke@mdc-berlin.de

Table of contents:

Appendix Figure S1	**FRET-based liposome binding assay.**	Page 2
Appendix Figure S2	**Rare binding events of soluble polymers to coated BPL liposomes.**	Page 3
Appendix Figure S3	**Analysis of the GBP1 coatomer.**	Page 4
Appendix Figure S4	**Image processing workflow for the membrane-bound GBP1 coatomer.**	Page 5
Appendix Figure S5	**Analysis of the soluble GBP1 polymers.**	Page 6
Appendix Figure S6	**Helix α4' protein structure.**	Page 7
Appendix Figure S7	**Intramolecular LG:GED contacts.**	Page 8
Appendix Figure S8	**Negative-stain TEM of GBP1 membrane-binding to BPL liposomes.**	Page 9

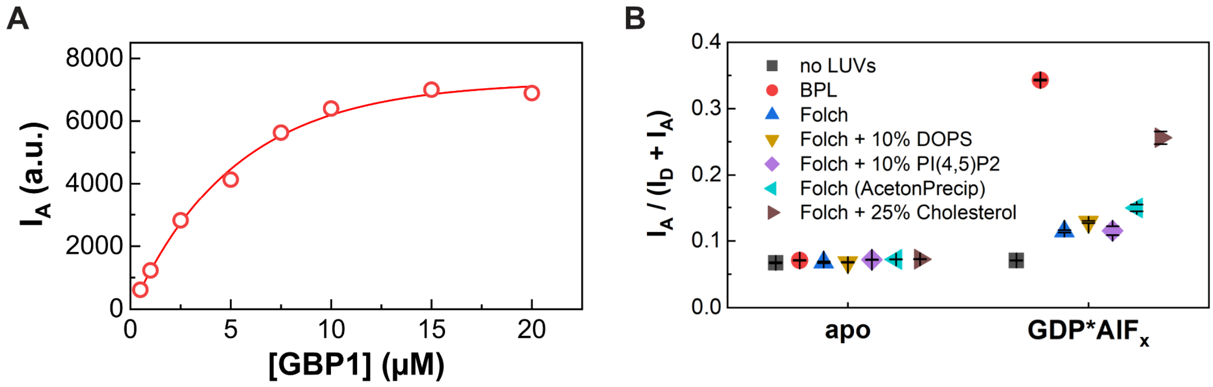

Appendix Figure S1. FRET-based liposome binding assay. (A) Concentration dependency of GBP1 binding to liposomes. Acceptor intensity of varying GBP1-Q577C-AF488 (donor) concentration at constant BPL liposome concentration supplemented with Liss Rhod PE (acceptor). **(B)** Ratiometric FRET efficiencies of GBP1-Q577C-AF488 (donor) incubated with indicated liposomes supplemented with Liss Rhod PE (acceptor) and indicated nucleotides. DOPS: 1,2-dioleoyl-sn-glycero-3-phospho-L-serine. PI(4,5)P_2: phosphatidylinositol-4,5-bisphosphate. AcetonPrecip: BPL extract is derived from a total lipid extract by precipitation with acetone (https://avantilipids.com/product/141101), which is not done in the Folch extraction procedure (Folch et al, 1957). Acetone precipitates phospholipids, while, for example, glycolipids and other simple lipids dissolve readily in acetone (Hanahan et al, 1951). Data from three independent replicates are shown as mean ± SD.

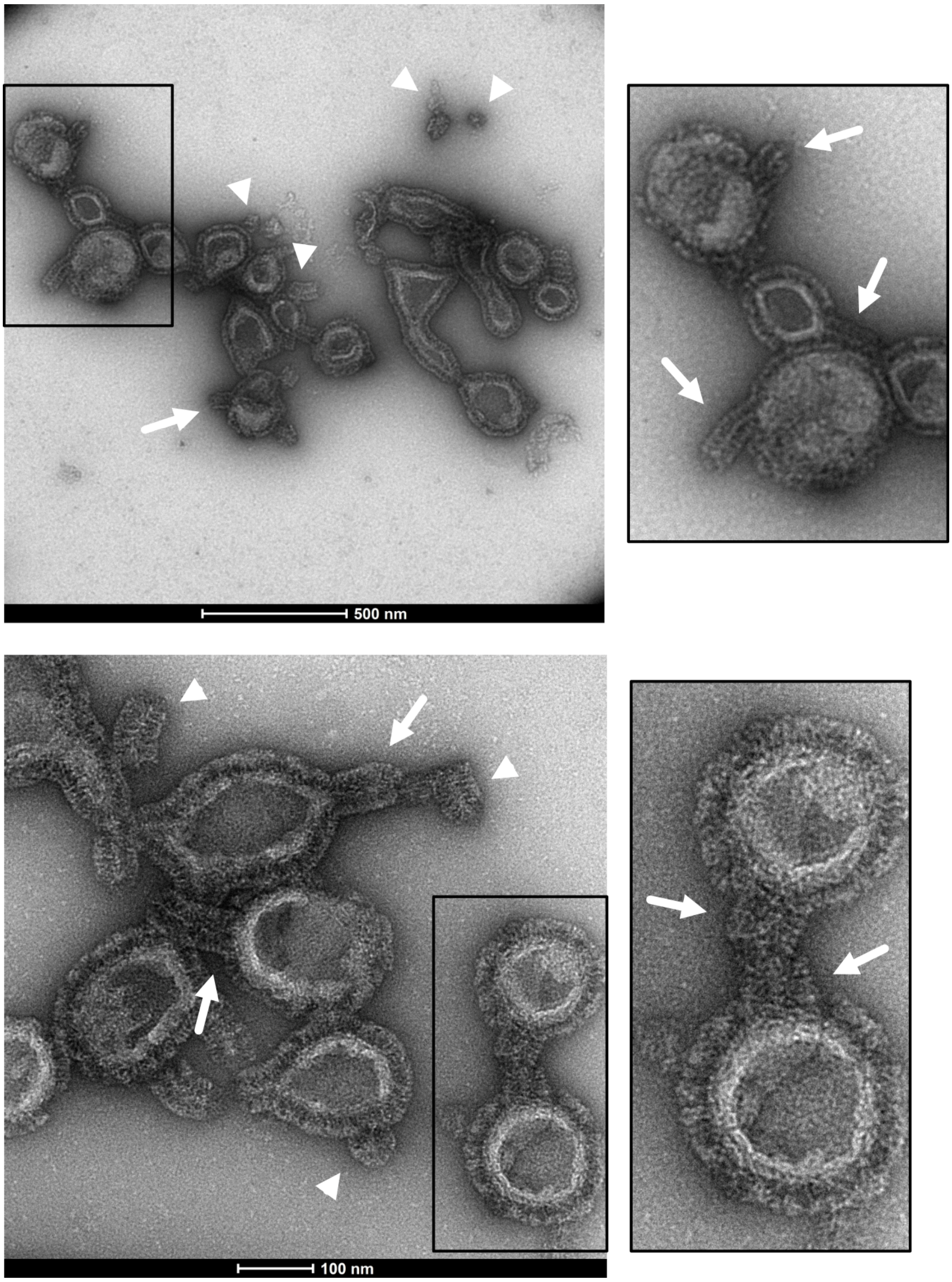

Appendix Figure S2. Rare binding events of soluble polymers to coated BPL liposomes. Oligomerization initiated with GDP•AlF$_x$. Arrows: attached polymers. Arrow heads: soluble polymers/disks. Scale bars as indicated.

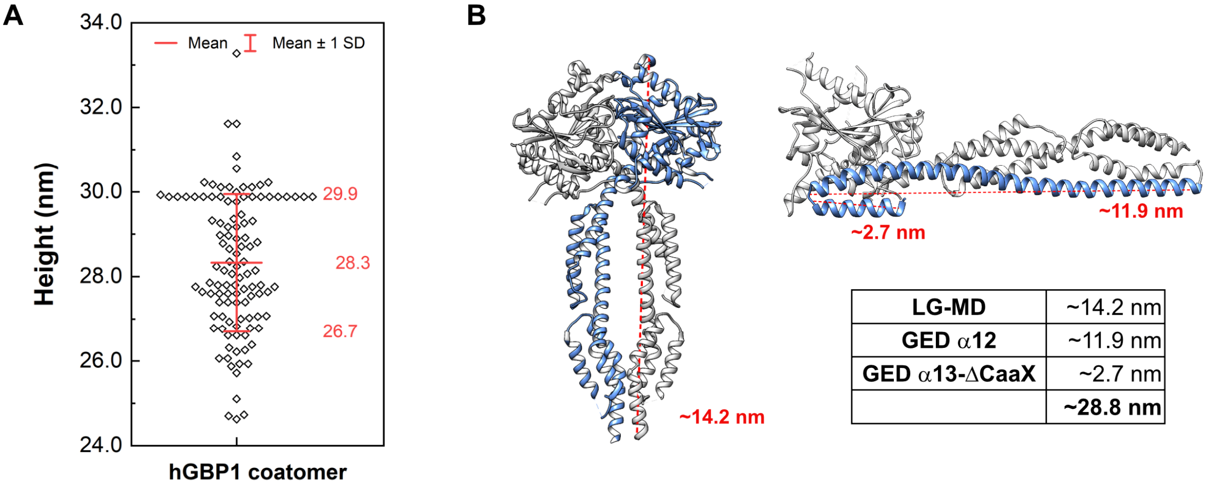

Appendix Figure S3. Analysis of the GBP1 coatomer. (A) Quantification of the coatomer height. 22 coated liposomes at 5 random positions were analyzed by measuring the distance from the lipid membrane to the very top of the coatomer (n=110). Average is shown as mean ± SD. **(B)** Theoretical length of outstretched GBP1. Left: GBP5-ΔGED dimer in complex with GDP•AlF_x (PDB 7e5a), Right: GBP1 in apo state (PDB 1dg3).

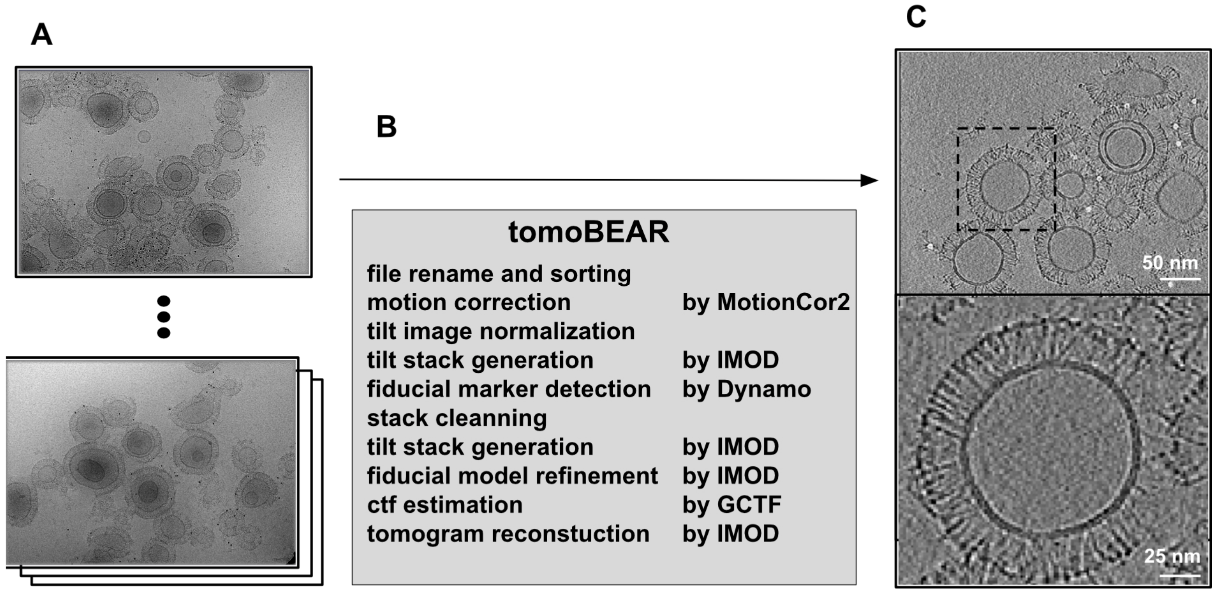

Appendix Figure S4. Image processing workflow for the membrane-bound GBP1 coatomer. (A) Raw images output from electron microscope. **(B)** Pipeline of processing and reconstruction executed by TomoBEAR. **(C)** Snapshot of the reconstructed tomogram (upper panel) and enlarged view of the marked GBP1 covered vesicle (lower panel).

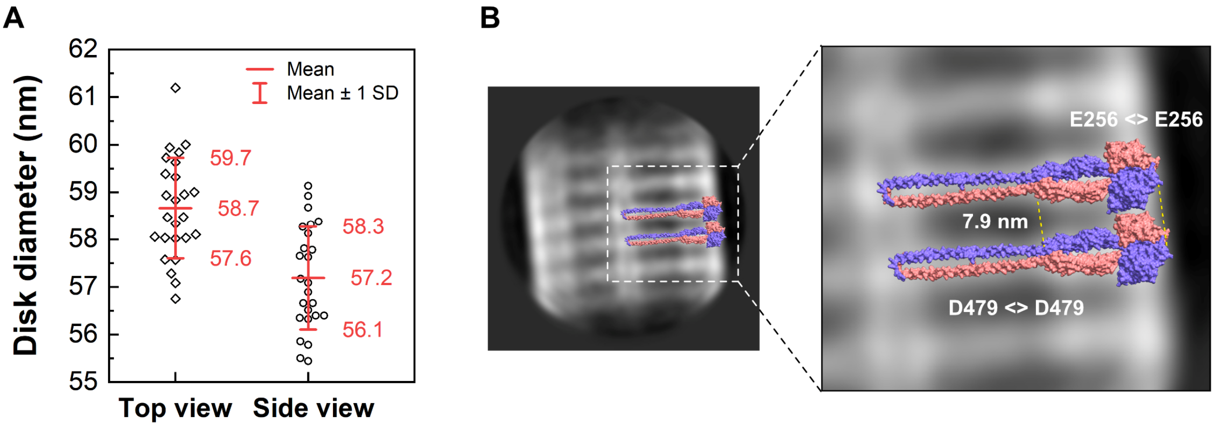

Appendix Figure S5. Analysis of the soluble GBP1 polymers. (A) Quantification of the diameter of disks in top view and stacks in side views (n=25). Averages are shown as mean ± SD. **(B)** 2D class of stacked polymeric disks modeled with the outstretched GBP1 dimer model. Distances between the LG domain and MD of two stacks were determined at C_α atoms of indicated amino acids.

A

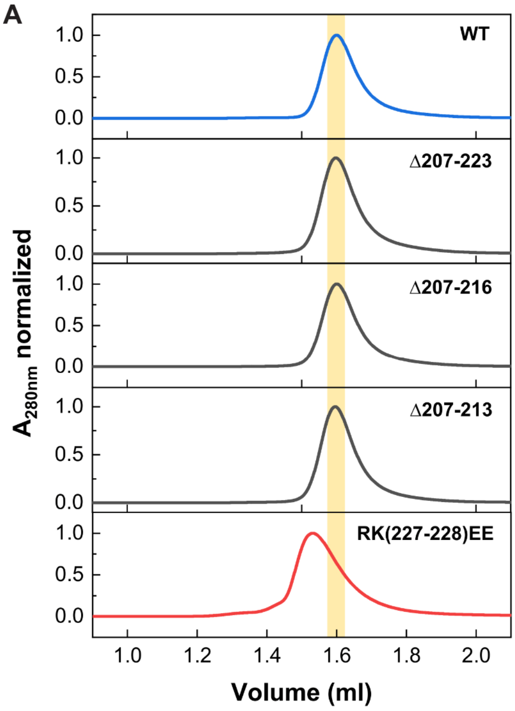

B

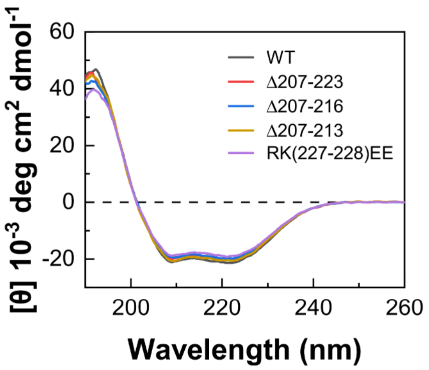

	WT	D207-223	D207-216	D207-213	RK(227-228)EE
**Helix**	65%	62%	61%	63%	58%
**Antiparallel**	2%	2%	2%	2%	3%
**Parallel**	5%	5%	6%	5%	6%
**Beta-Turn**	12%	12%	13%	12%	13%
**Random Coil**	19%	20%	21%	20%	22%

Appendix Figure S6. Helix α4’ protein structure. (A) Analytical SEC. Wild-type GBP1 and the open GBP1 mutant RK(227-228)EE were used as references for changes in the overall protein architecture. The peak of the monomeric wild-type GBP1 species is highlighted in yellow for comparison. **(B)** Circular dichroism. Top: CD spectra normalized according to the cell path length, protein concentration and number of amino acid residues. Bottom: Secondary-structure determination using CDNN 2.1.

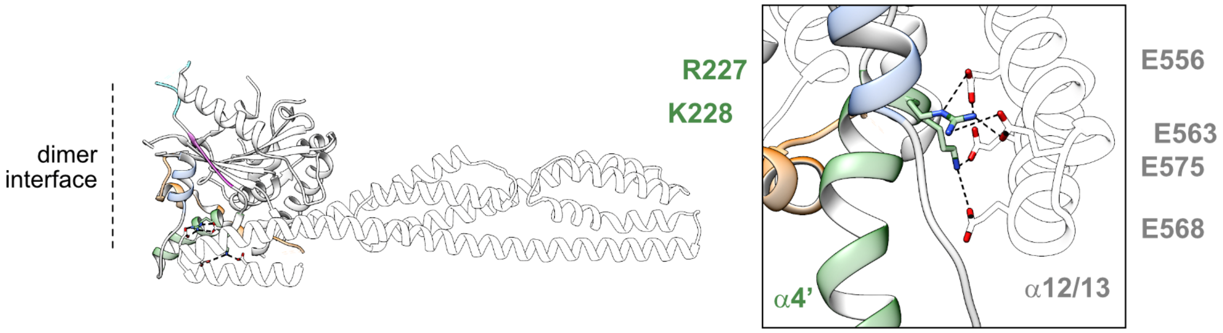

Appendix Figure S7. Intramolecular LG:GED contacts. The crystal structure of full-length GBP1 in the closed monomeric state (apo, PDB 1dg3) shows a salt bridge network between R227/K228 of helix α4' (LG) and the four glutamate residues 556, 563, 568, and 575 of helix α12/13 (GED), stabilizing the closed conformation.

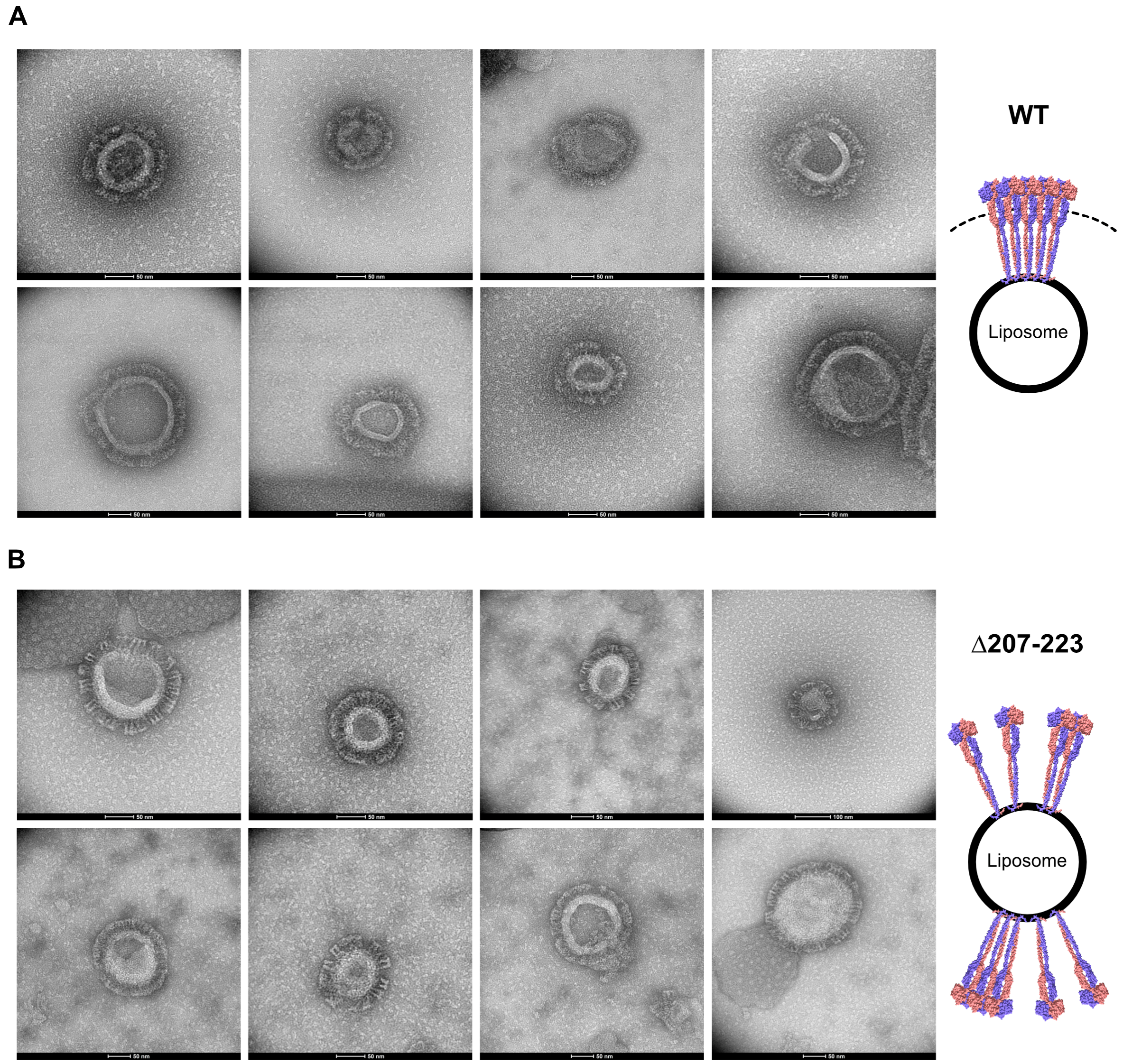

Appendix Figure S8. Negative-stain TEM of GBP1 membrane-binding to BPL liposomes. Representative micrographs of **(A)** wild-type (WT) and **(B)** Δ207-223 variant used in the blinded quantification experiment of a uniform protein coat. Scheme: uniform (WT) vs. patched (Δ207-223) protein coat. Scale bars as indicated.
