# Supplementary material for: Structural insights into the activation mechanism of antimicrobial GBP1
Source: EMBO J. 2024 Jan 24;43(4):615–36. doi: 10.1038/s44318-023-00023-y (PMC10897159; doi:10.1038/s44318-023-00023-y)
Supplement: Supplementary file 2 — Movie EV1 [file 44318_2023_23_MOESM2_ESM.zip › EMBOJ-2023-115158_MovieEV1_Legend.docx]

**Movie EV1. Model of the outstretched GBP1 dimer and zoom into the dimeric LG domain.**

The LG domain is in complex with the GTP analogue GMPPNP (PDB 2bc9) and mediates lateral LG:LG interactions within the oligomer.
