# Supplementary material for: Structural insights into the activation mechanism of antimicrobial GBP1
Source: EMBO J. 2024 Jan 24;43(4):615–36. doi: 10.1038/s44318-023-00023-y (PMC10897159; doi:10.1038/s44318-023-00023-y)
Supplement: Supplementary file 3 — Movie EV2 [file 44318_2023_23_MOESM3_ESM.zip › EMBOJ-2023-115158_MovieEV2_Legend.docx]

**Movie EV2. *In vitro* binding assay of GBP1 helix α4’ variants.**

Time-lapse confocal microscopy of GBP1 wild-type or helix α4’ variants (magenta) targeting pathogenic *E. coli* (green).
