# Supplementary material for: Structural insights into the activation mechanism of antimicrobial GBP1
Source: EMBO J. 2024 Jan 24;43(4):615–36. doi: 10.1038/s44318-023-00023-y (PMC10897159; doi:10.1038/s44318-023-00023-y)
Supplement: Supplementary file 4 — Movie EV3 [file 44318_2023_23_MOESM4_ESM.zip › EMBOJ-2023-115158_MovieEV3_Legend.docx]

**Movie EV3. Morph of the GBP1 LG domains in different nucleotide states.**

Structural rearrangement of the guanine cap and helix α3 and α4’ upon nucleotide hydrolysis are displayed. PDB accession codes: apo (1dg3), GMP (2d4h), GMP•AlF_x_ (2b8w), GDP•AlF_x_ (2b92), GMPPNP (2bc9).
