# Supplementary material for: Structural insights into the activation mechanism of antimicrobial GBP1
Source: EMBO J. 2024 Jan 24;43(4):615–36. doi: 10.1038/s44318-023-00023-y (PMC10897159; doi:10.1038/s44318-023-00023-y)
Supplement: Supplementary file 5 — Movie EV4 [file 44318_2023_23_MOESM5_ESM.zip › EMBOJ-2023-115158_MovieEV4_Legend.docx]

**Movie EV4. Morph of the coordinated helix α3-α4’ movement in different nucleotide states.**

Structural rearrangement of helix α3 and α4’ upon nucleotide hydrolysis are displayed and the salt bridge between D199 and K234 is shown. PDB accession codes: apo (1dg3), GMP (2d4h), GMP•AlF_x_ (2b8w), GDP•AlF_x_ (2b92), GMPPNP (2bc9).
