# Supplementary figures and images for: Structural insights into the activation mechanism of antimicrobial GBP1
Source: EMBO J. 2024 Jan 24;43(4):615–36. doi: 10.1038/s44318-023-00023-y (PMC10897159; doi:10.1038/s44318-023-00023-y)

SourceData Fig. 1B

no lipids

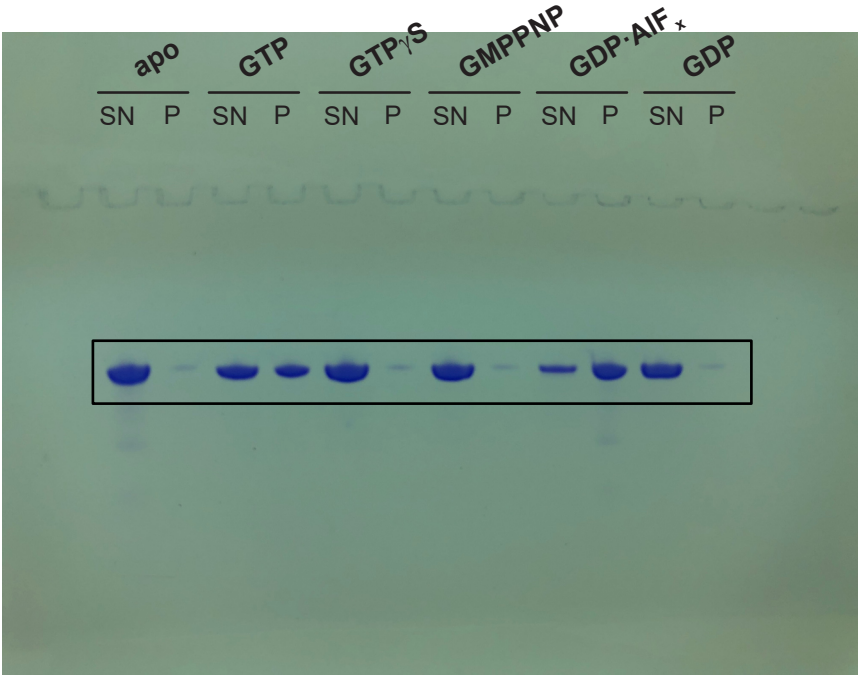

BPL

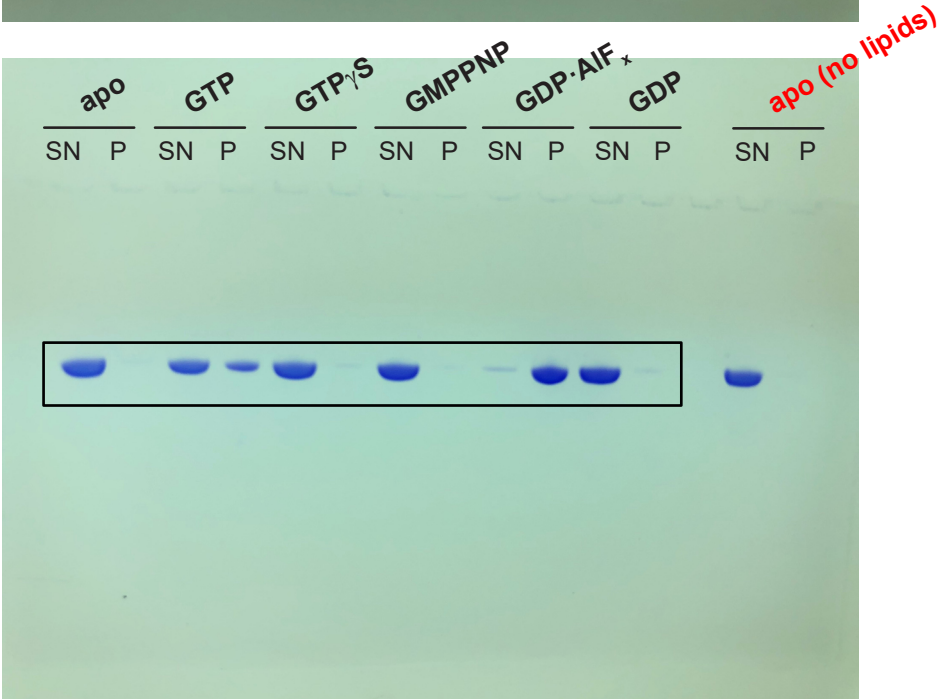

Folch

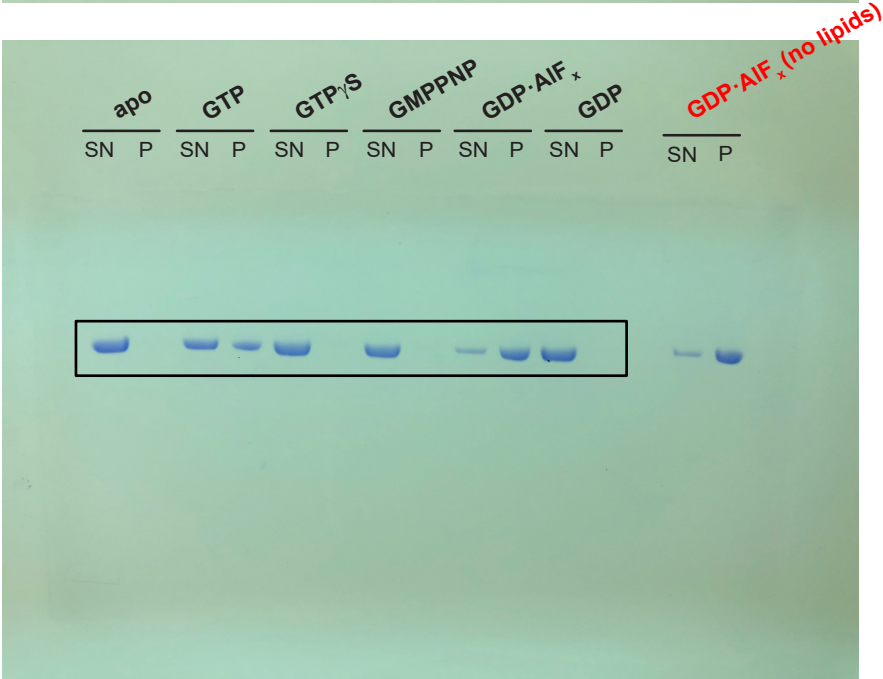

Supplement: Supplementary file 6 — Source Data Fig. 1 [file 44318_2023_23_MOESM6_ESM.zip › Figure 1/1B/SourceData_1B_co-sedimentation assay of WT varying nucleotides and lipids.pdf]

SourceData Fig. 1D

BPL +GTP

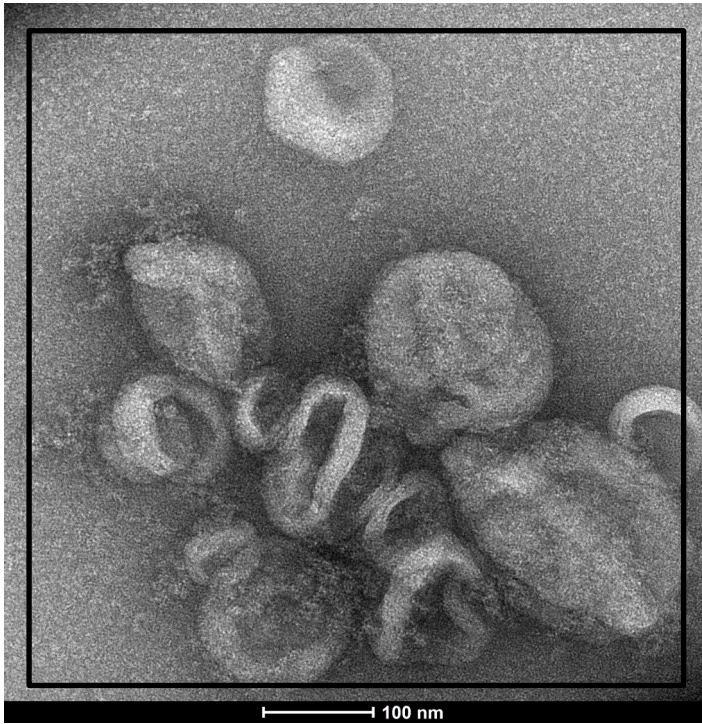

BPL +GDP-AIFx

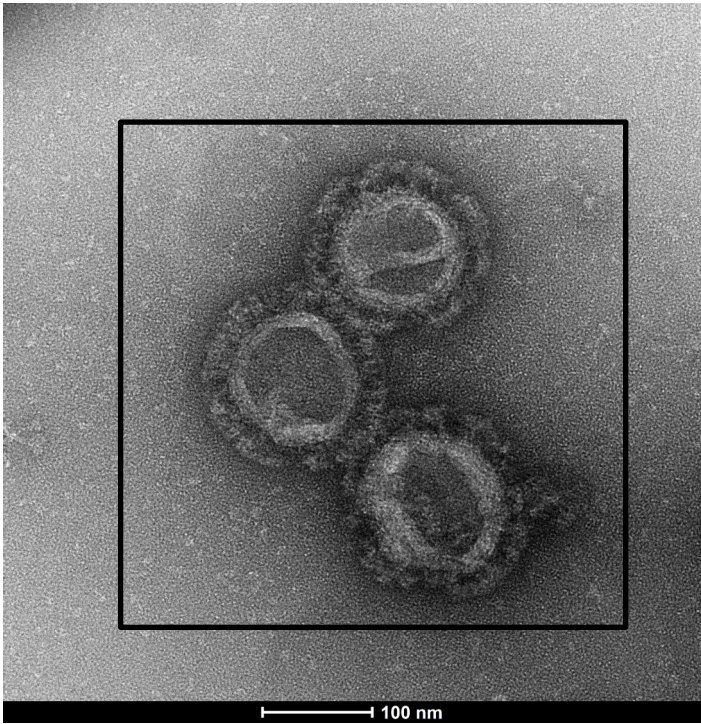

Folch +GTP

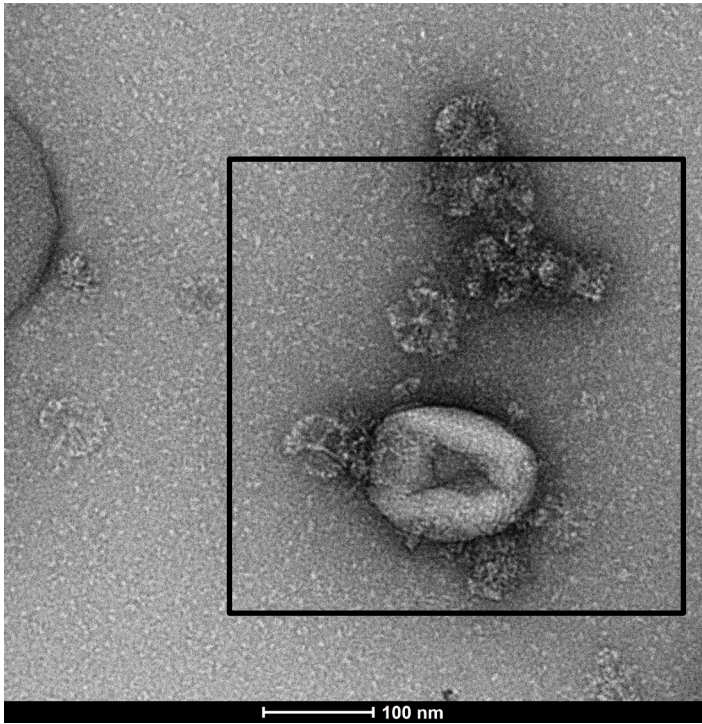

Folch +GDP-AIFx

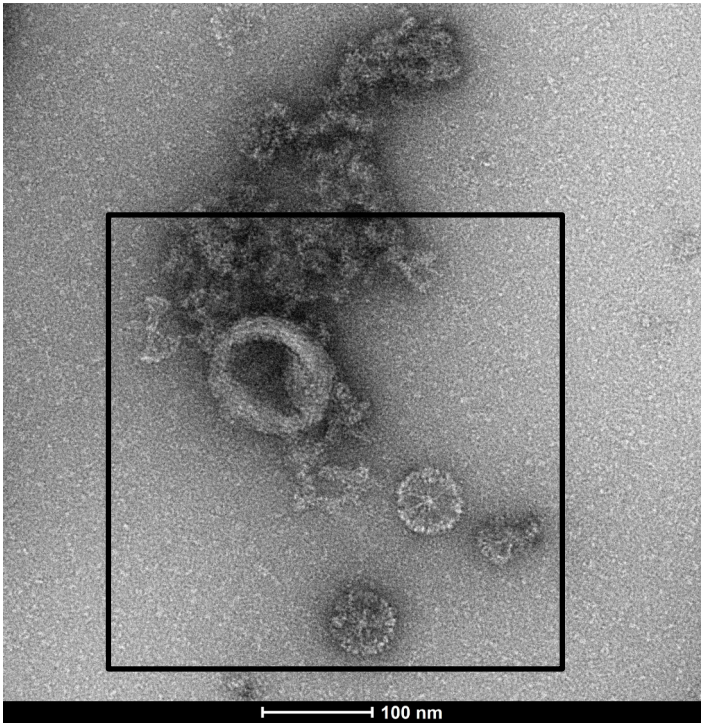

Supplement: Supplementary file 6 — Source Data Fig. 1 [file 44318_2023_23_MOESM6_ESM.zip › Figure 1/1D/SourceData_1D_negative-stain TEM of WT varying nucleotides and lipids.pdf]

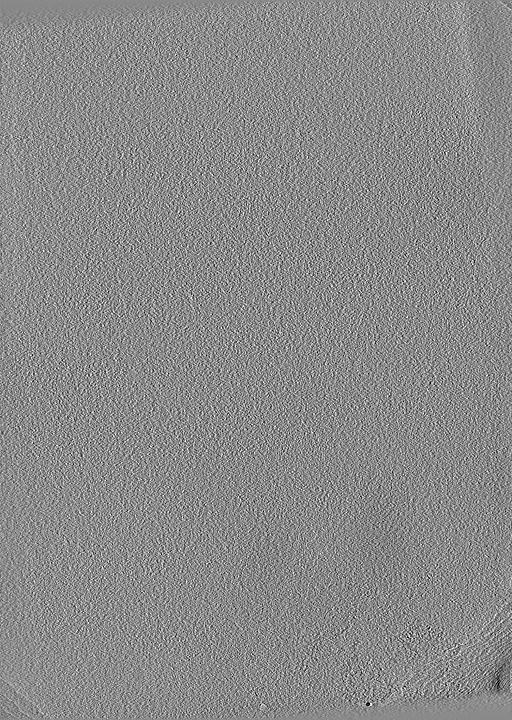

Supplement: Supplementary file 6 — Source Data Fig. 1 [file 44318_2023_23_MOESM6_ESM.zip › Figure 1/1E/SourceData_1E_tomogram of WT bound to BPL.tif]

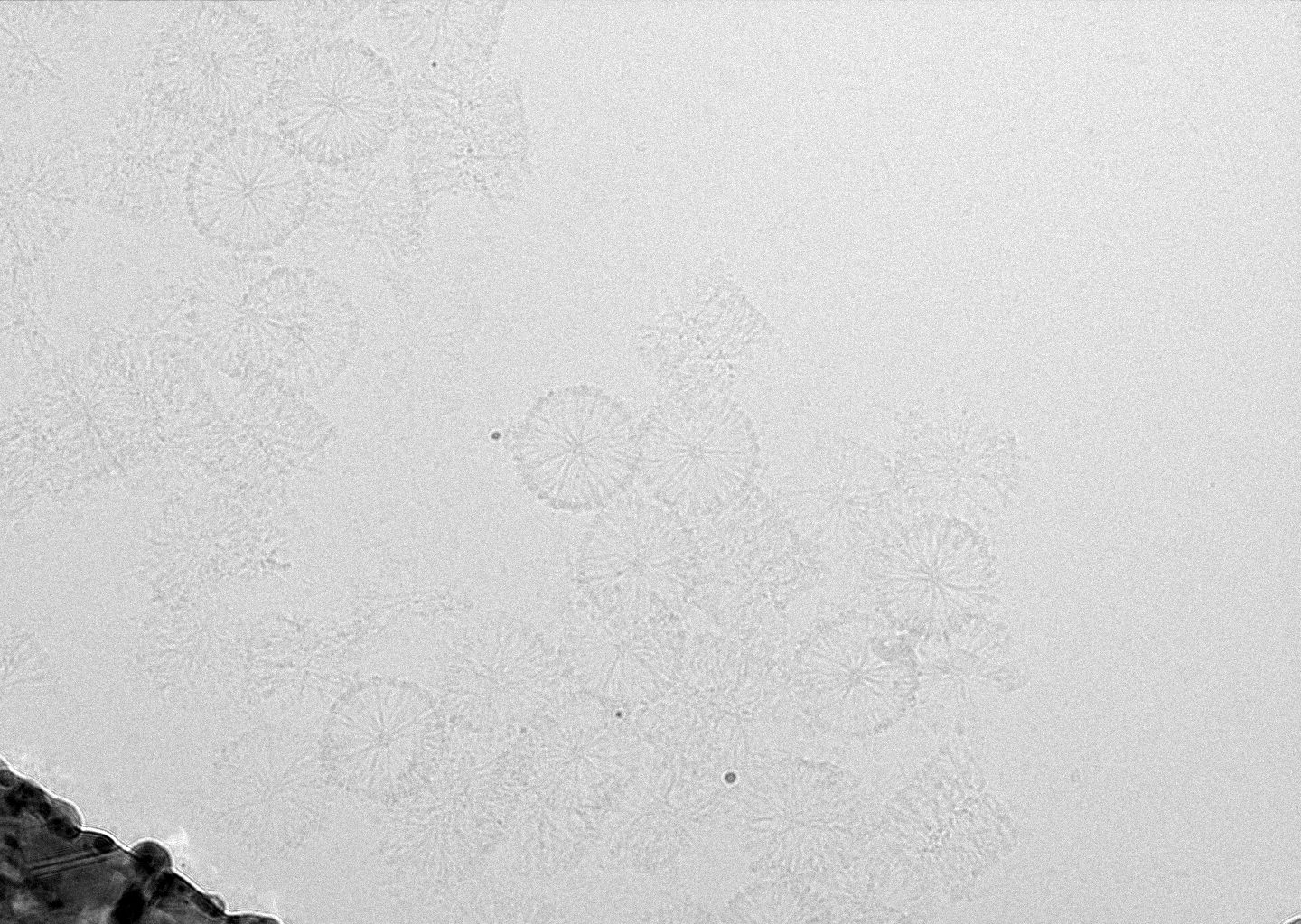

Supplement: Supplementary file 6 — Source Data Fig. 1 [file 44318_2023_23_MOESM6_ESM.zip › Figure 1/1H/SourceData_1H_FoilHole_27163014_Data_27160047_27160049_20230227_193203.tif]

**SourceData Fig. 2C**

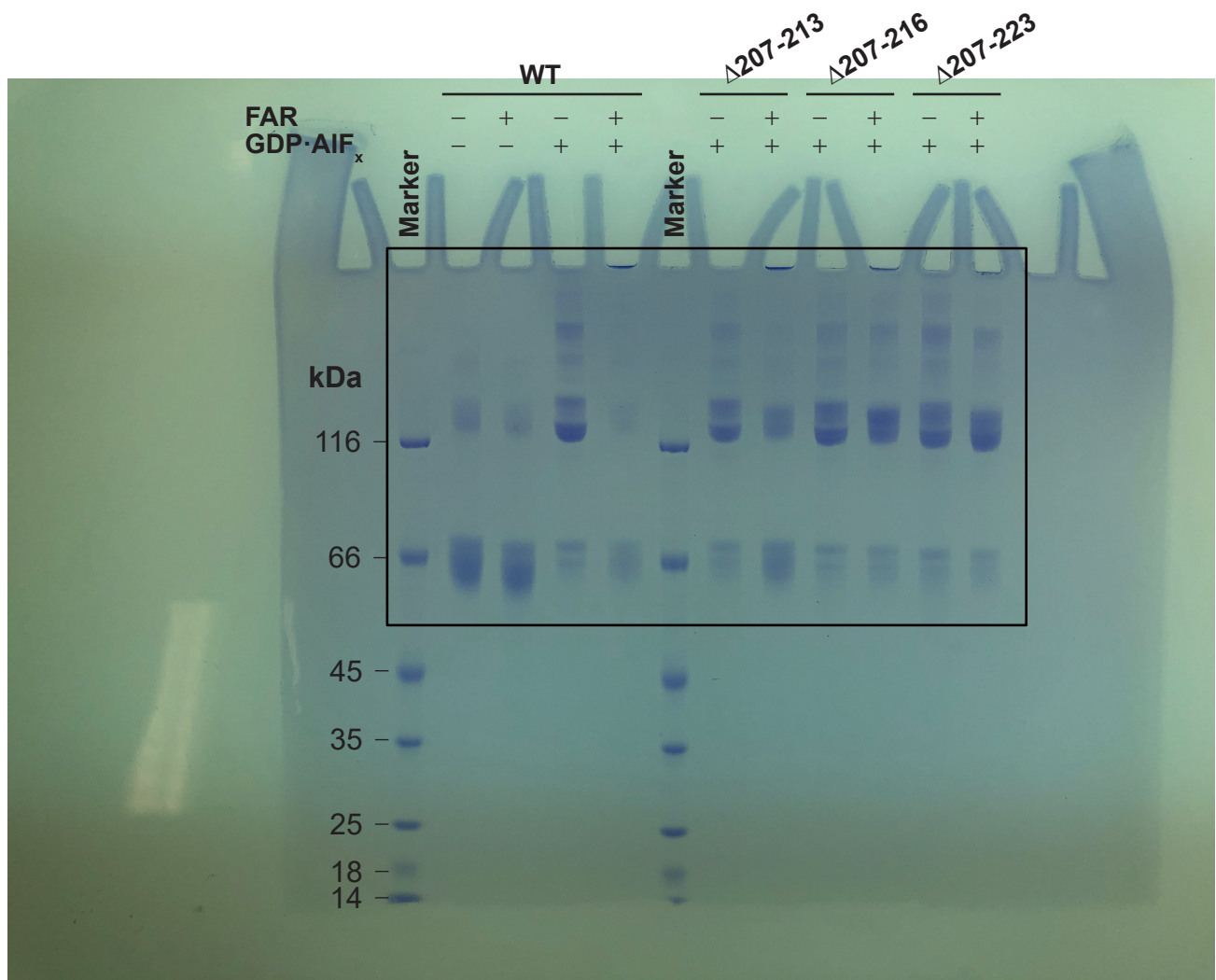

Supplement: Supplementary file 7 — Source Data Fig. 2 [file 44318_2023_23_MOESM7_ESM.zip › Figure 2/2C/SourceData_2C_crosslinking assay of a4' mutants.pdf]

SourceData Fig. 2E

no lipids

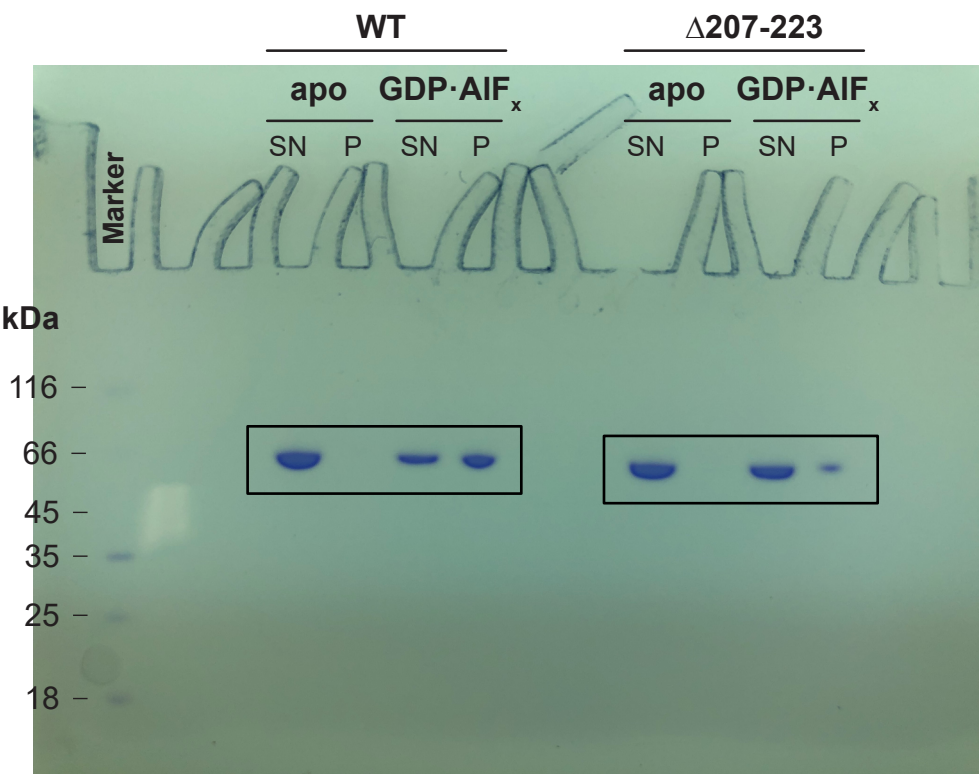

+ BPL

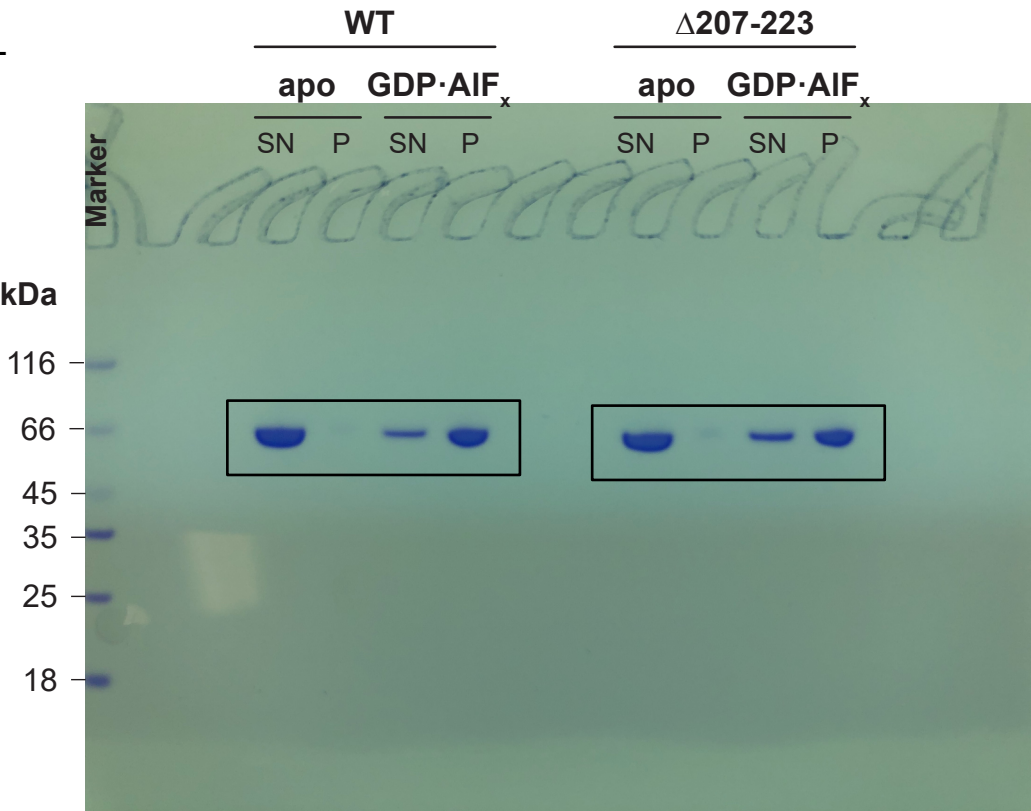

Supplement: Supplementary file 7 — Source Data Fig. 2 [file 44318_2023_23_MOESM7_ESM.zip › Figure 2/2E/SourceData_2E_co-sedimentation assay of D207-223.pdf]

SourceData Fig. 2F

WT +GDP-AIFx

large field of view

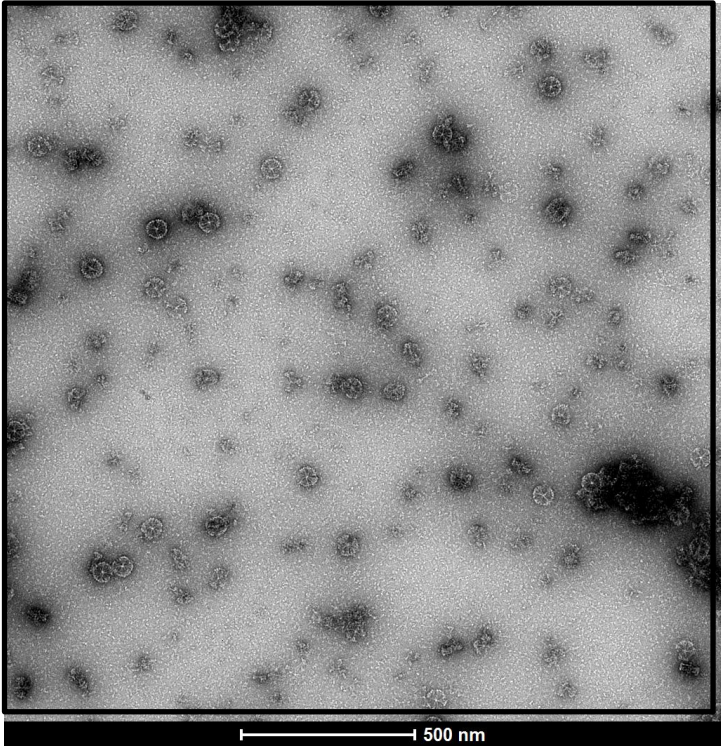

zoom

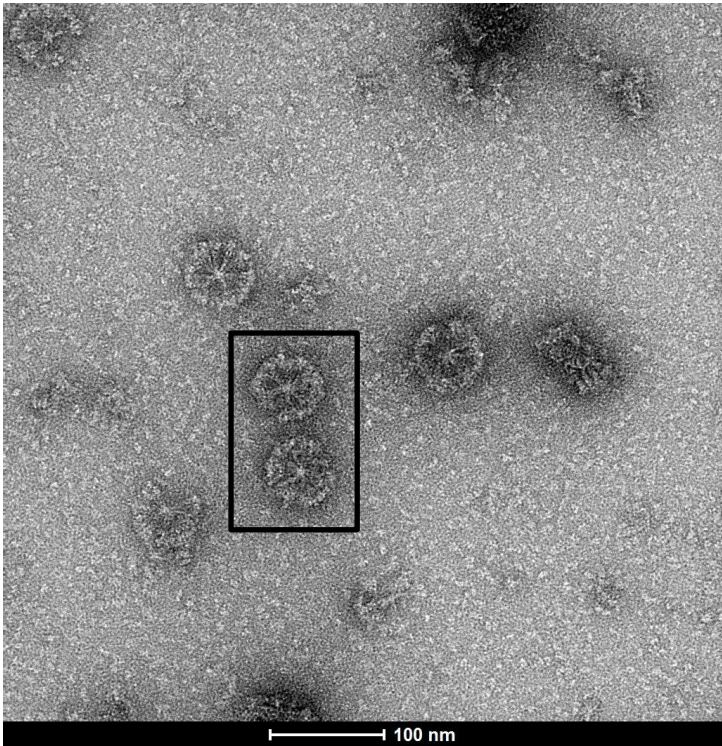

$\Delta$ 207-223 +GDP-AIFx

large field of view

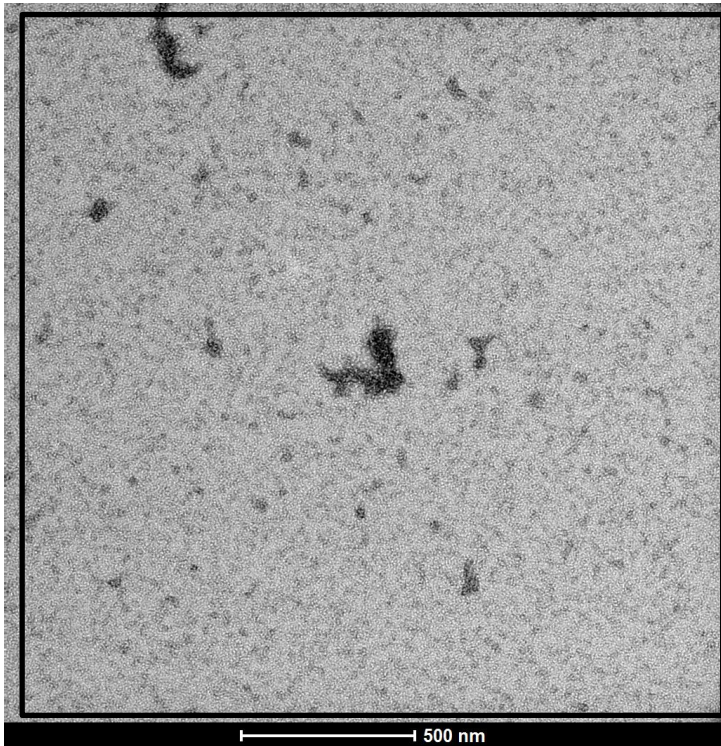

zoom

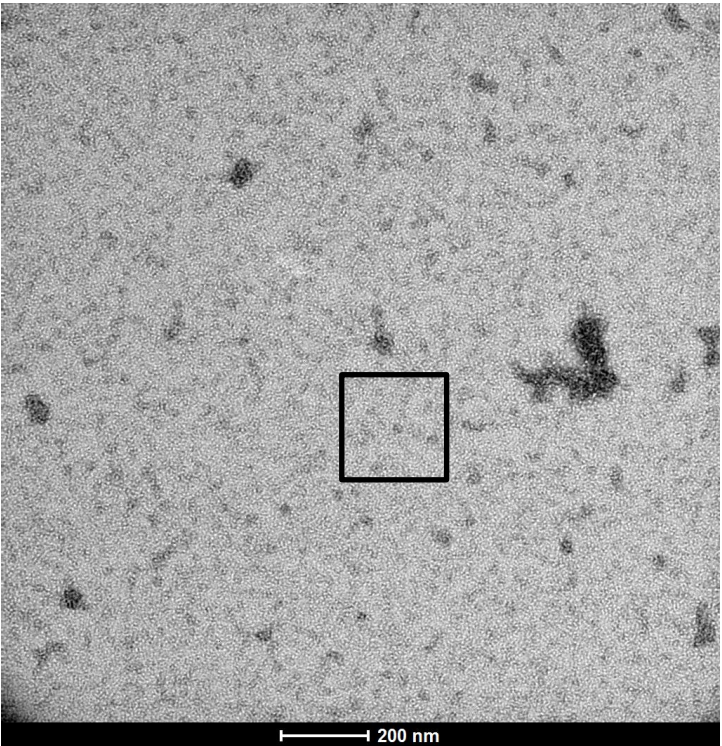

Supplement: Supplementary file 7 — Source Data Fig. 2 [file 44318_2023_23_MOESM7_ESM.zip › Figure 2/2F/SourceData_2F_negative-stain TEM of WT vs D207-223 polymerization.pdf]

SourceData Fig. 2G

WT +BPL +GDP-AIFx

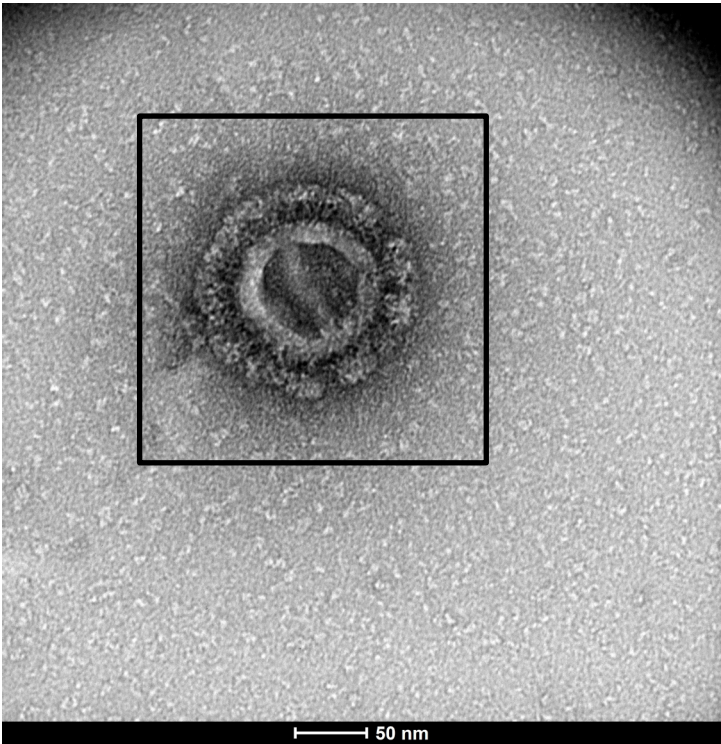

$\Delta$ 207-223 +BPL +GDP-AIFx

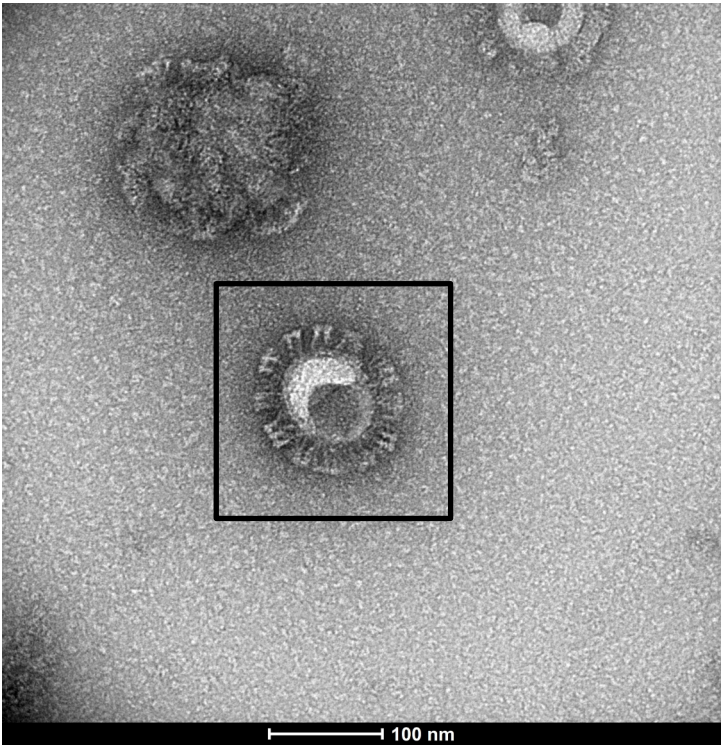

Supplement: Supplementary file 7 — Source Data Fig. 2 [file 44318_2023_23_MOESM7_ESM.zip › Figure 2/2G/SourceData_2G_negative-stain TEM of WT vs D207-223 on BPL.pdf]

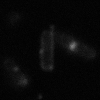

Supplement: Supplementary file 8 — Source Data Fig. 3 [file 44318_2023_23_MOESM8_ESM.zip › Figure 3/3F/207-213_crop.tif]

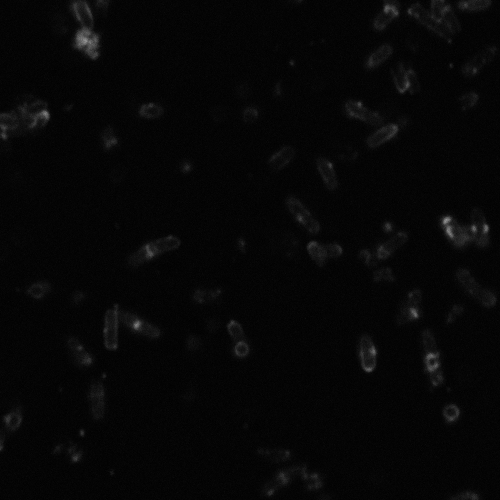

Supplement: Supplementary file 8 — Source Data Fig. 3 [file 44318_2023_23_MOESM8_ESM.zip › Figure 3/3F/207-213_detail.tif]

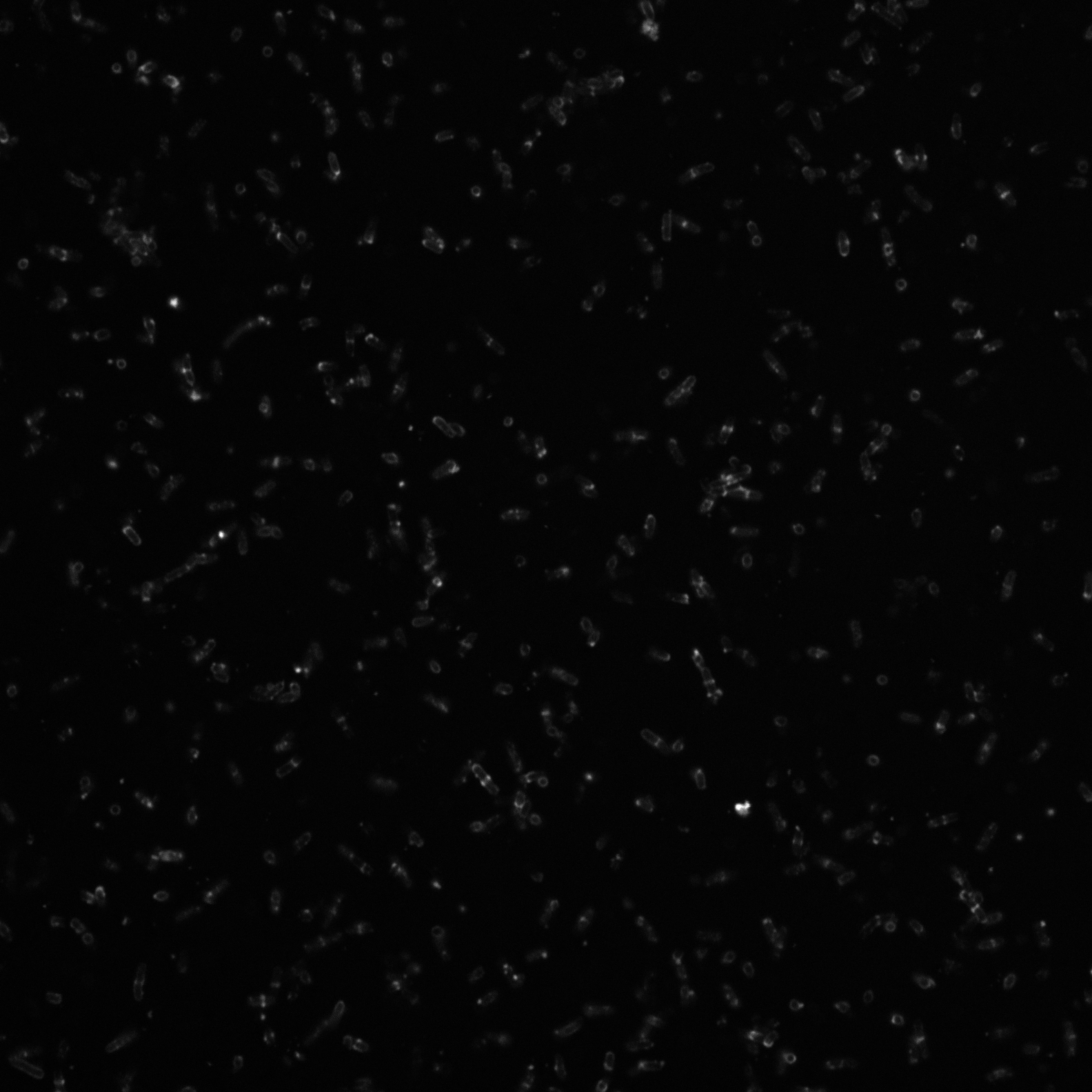

Supplement: Supplementary file 8 — Source Data Fig. 3 [file 44318_2023_23_MOESM8_ESM.zip › Figure 3/3F/207-213_original.tif]

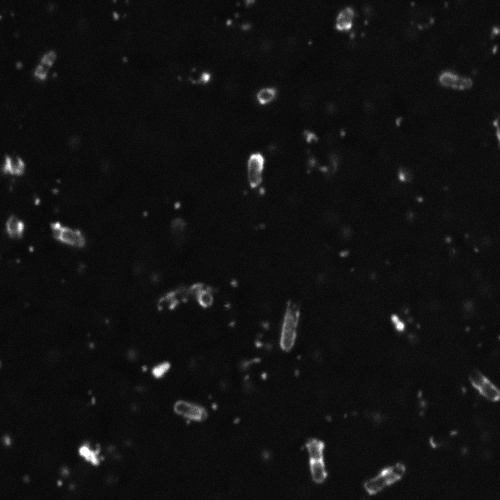

Supplement: Supplementary file 8 — Source Data Fig. 3 [file 44318_2023_23_MOESM8_ESM.zip › Figure 3/3F/207-216_crop.tif]

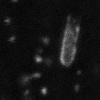

Supplement: Supplementary file 8 — Source Data Fig. 3 [file 44318_2023_23_MOESM8_ESM.zip › Figure 3/3F/207-216_detail.tif]

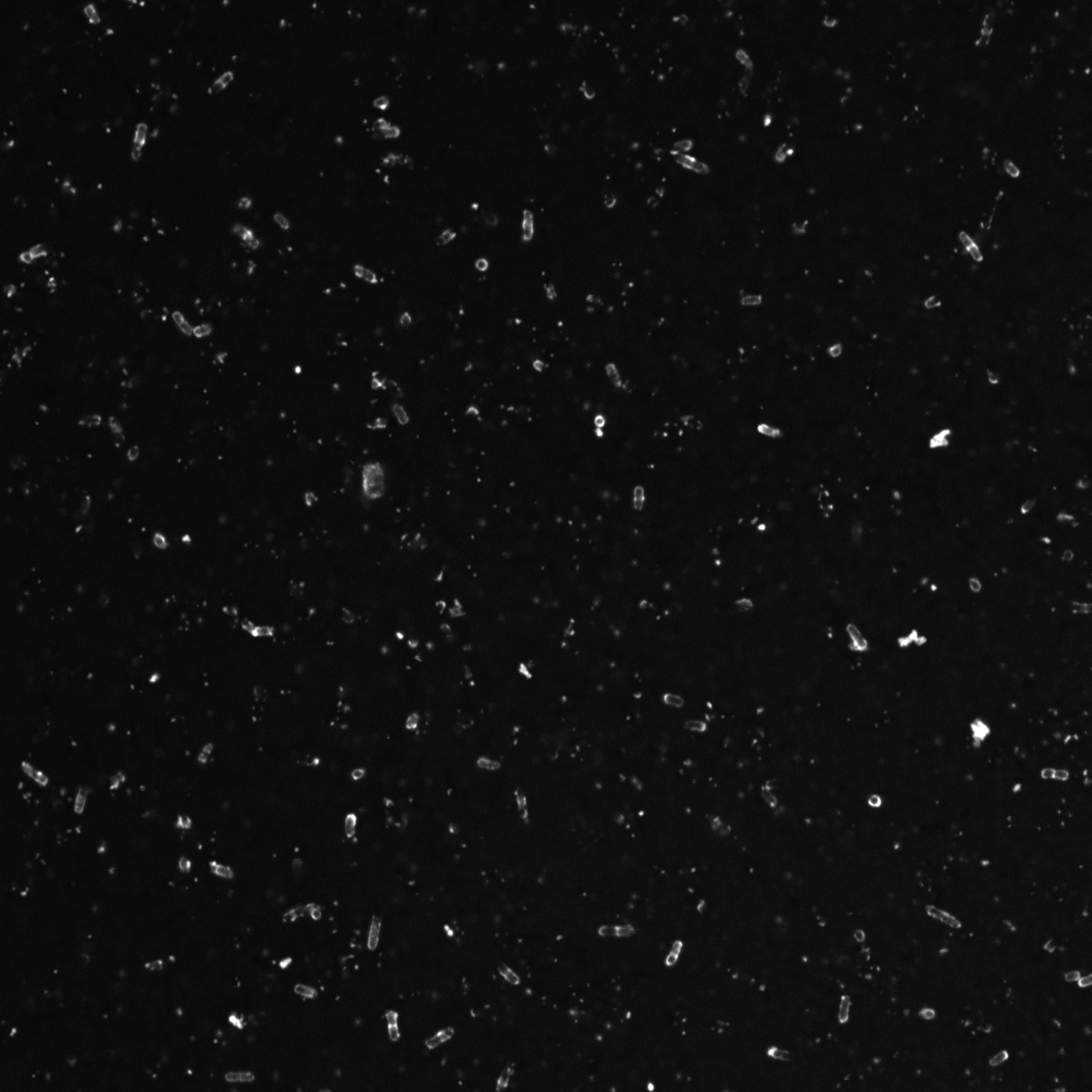

Supplement: Supplementary file 8 — Source Data Fig. 3 [file 44318_2023_23_MOESM8_ESM.zip › Figure 3/3F/207-216_original.tif]

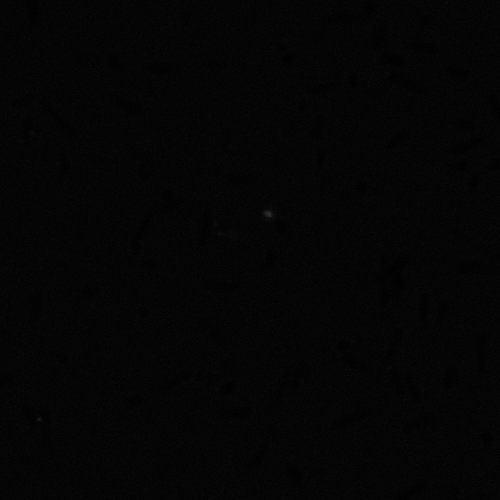

Supplement: Supplementary file 8 — Source Data Fig. 3 [file 44318_2023_23_MOESM8_ESM.zip › Figure 3/3F/207-223_crop.tif]

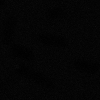

Supplement: Supplementary file 8 — Source Data Fig. 3 [file 44318_2023_23_MOESM8_ESM.zip › Figure 3/3F/207-223_detail.tif]

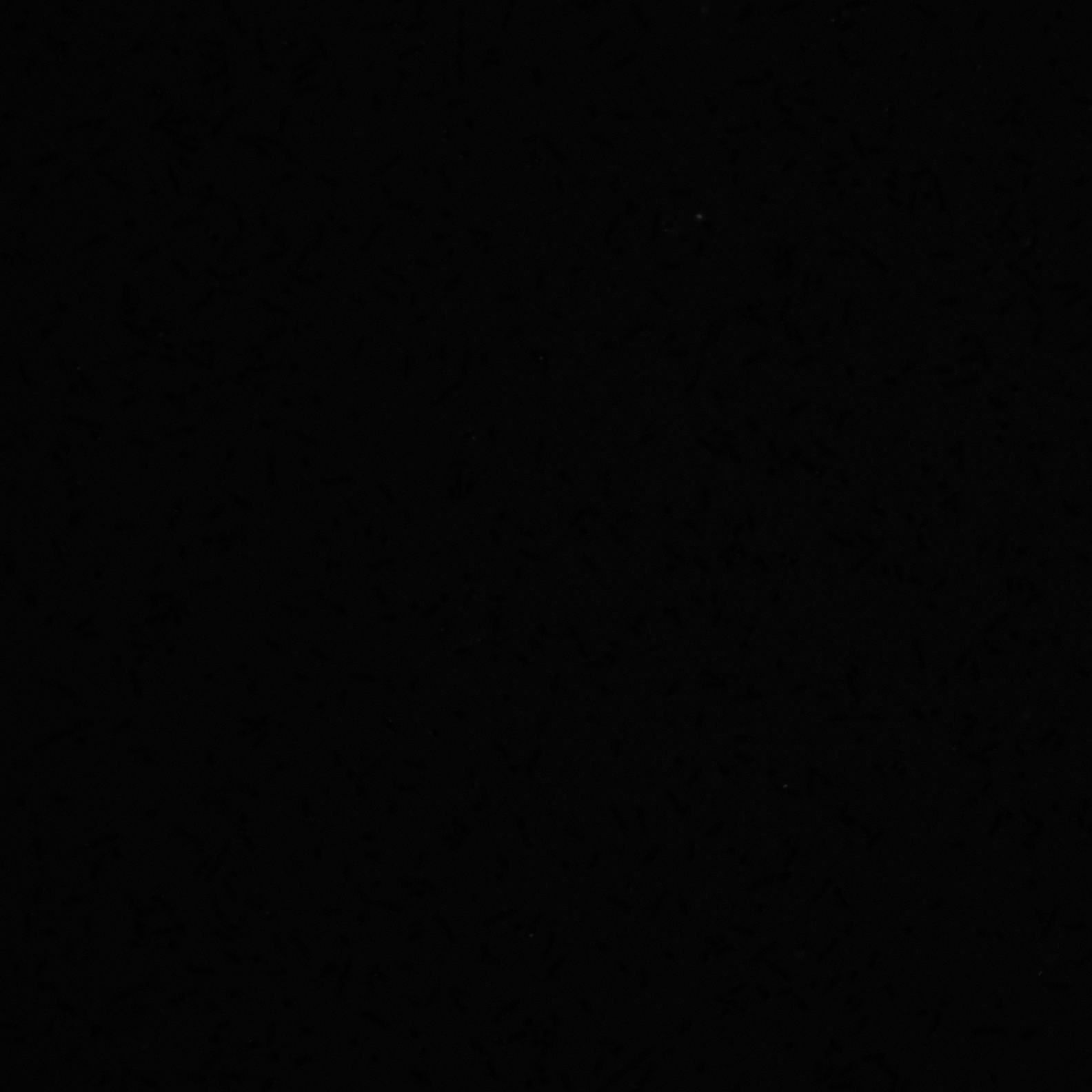

Supplement: Supplementary file 8 — Source Data Fig. 3 [file 44318_2023_23_MOESM8_ESM.zip › Figure 3/3F/207-223_original.tif]

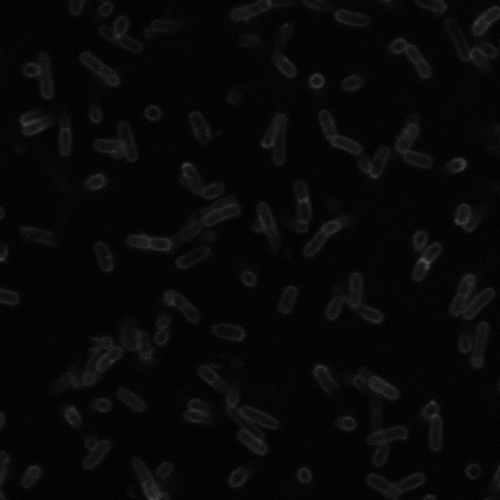

Supplement: Supplementary file 8 — Source Data Fig. 3 [file 44318_2023_23_MOESM8_ESM.zip › Figure 3/3F/WT_crop.tif]

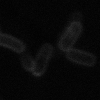

Supplement: Supplementary file 8 — Source Data Fig. 3 [file 44318_2023_23_MOESM8_ESM.zip › Figure 3/3F/WT_detail.tif]

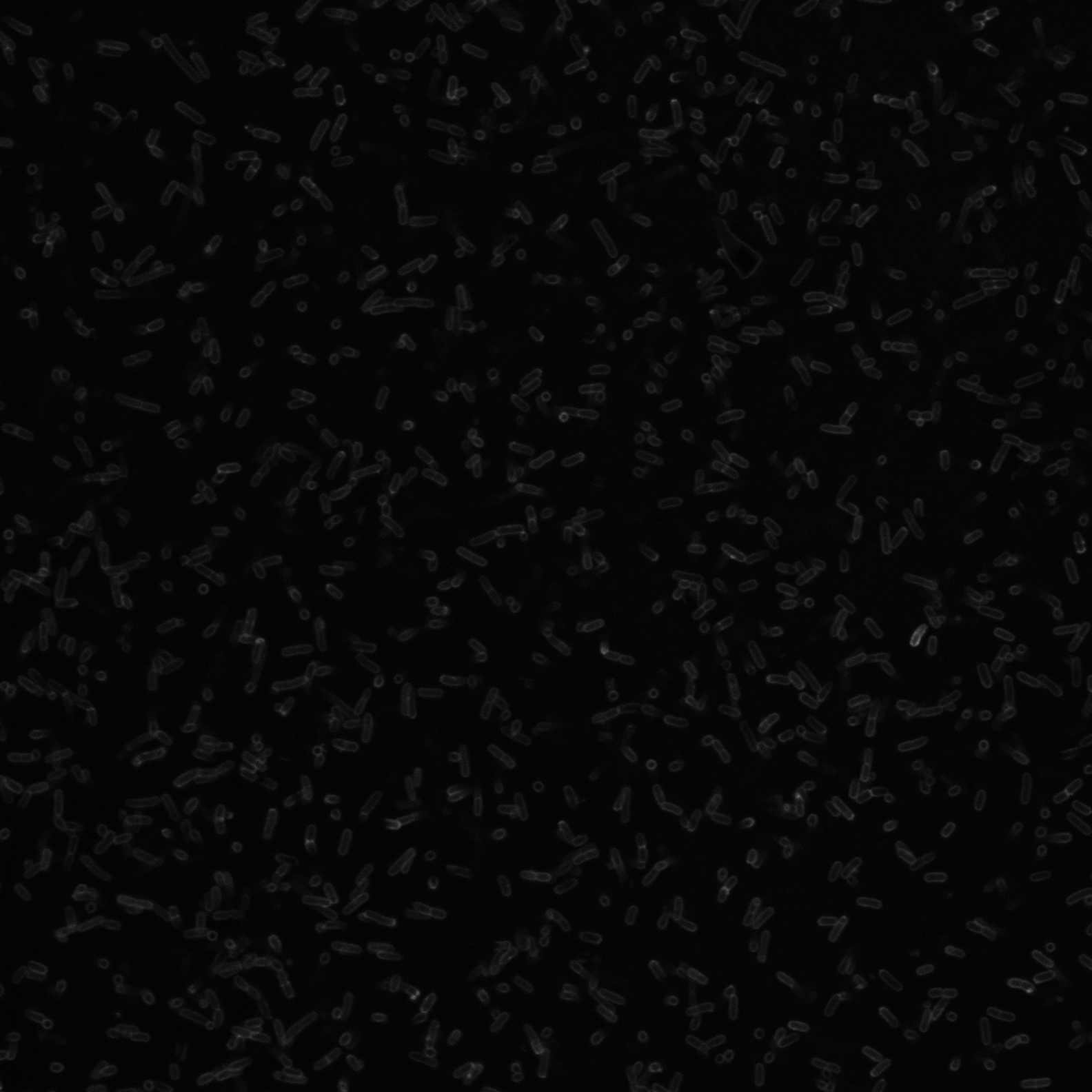

Supplement: Supplementary file 8 — Source Data Fig. 3 [file 44318_2023_23_MOESM8_ESM.zip › Figure 3/3F/WT_original.tif]

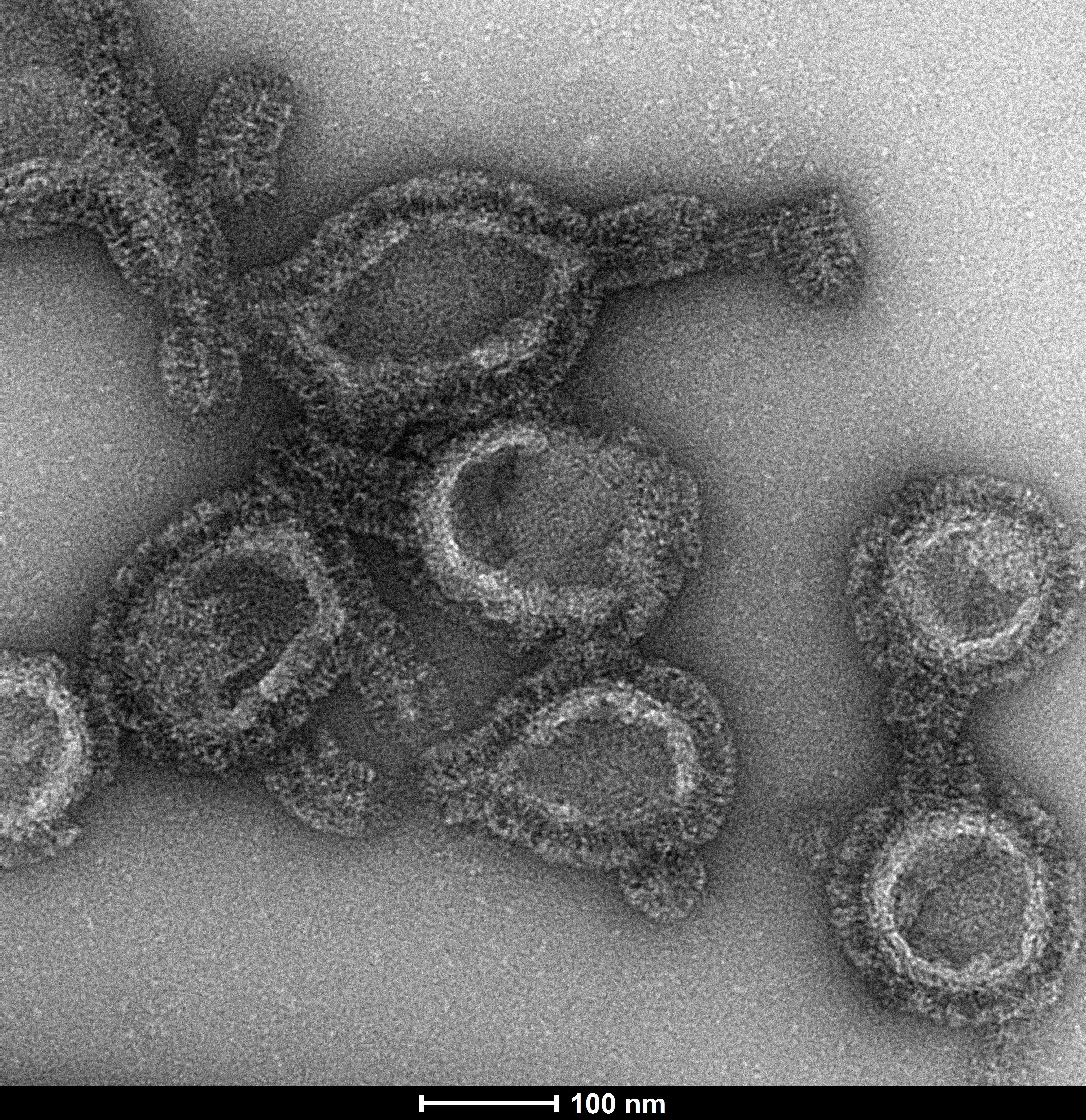

Supplement: Supplementary file 10 — Figures EV and Appendix Source Data [file 44318_2023_23_MOESM10_ESM.zip › Appendix Figure S2/211130_K5_BPL_GDP-AlF_1to10__0006.jpg]

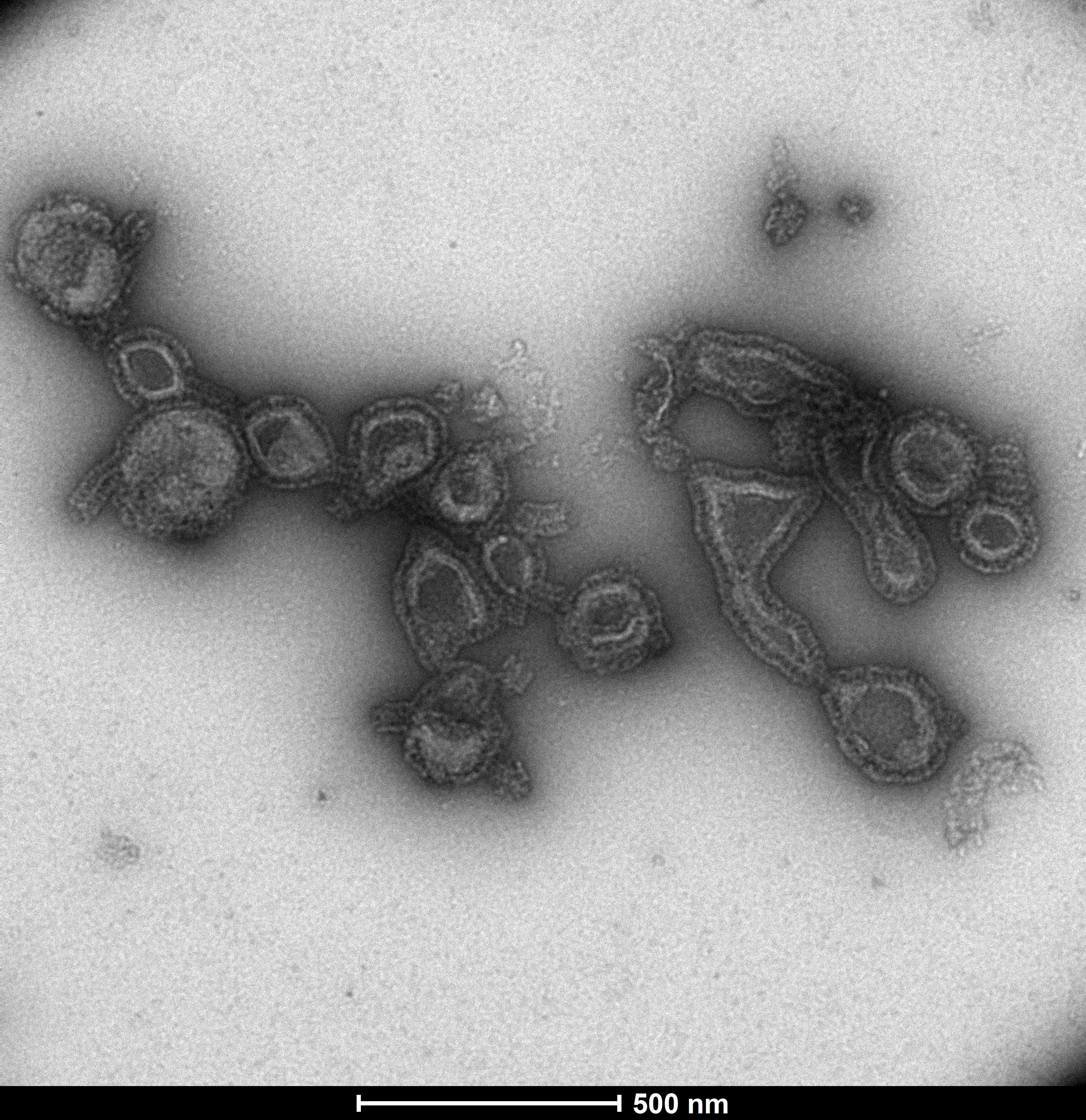

Supplement: Supplementary file 10 — Figures EV and Appendix Source Data [file 44318_2023_23_MOESM10_ESM.zip › Appendix Figure S2/211130_K5_BPL_GDP-AlF_1to10__0013.jpg]

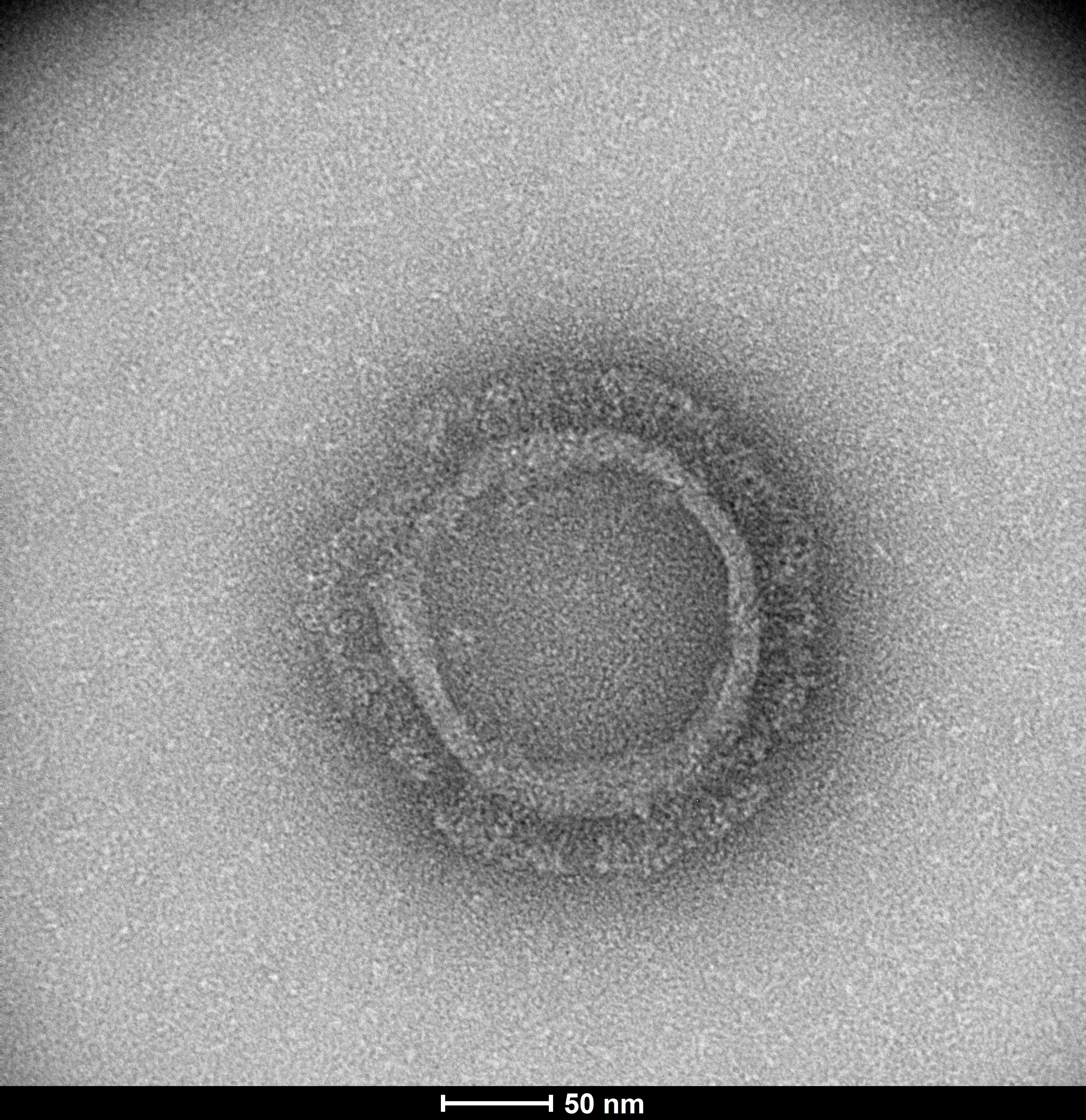

Supplement: Supplementary file 10 — Figures EV and Appendix Source Data [file 44318_2023_23_MOESM10_ESM.zip › Appendix Figure S8/S8A/12.jpg]

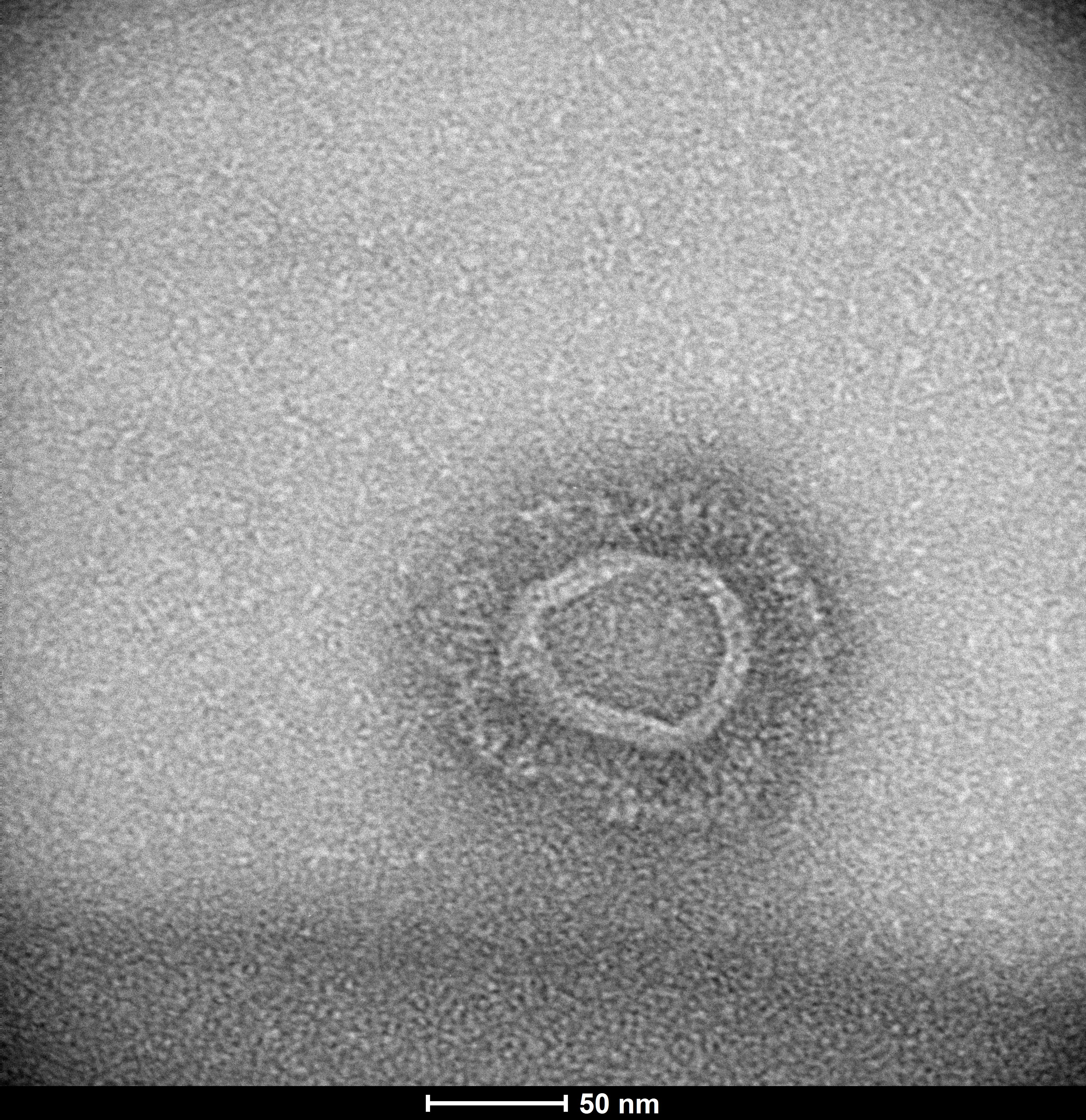

Supplement: Supplementary file 10 — Figures EV and Appendix Source Data [file 44318_2023_23_MOESM10_ESM.zip › Appendix Figure S8/S8A/15.jpg]

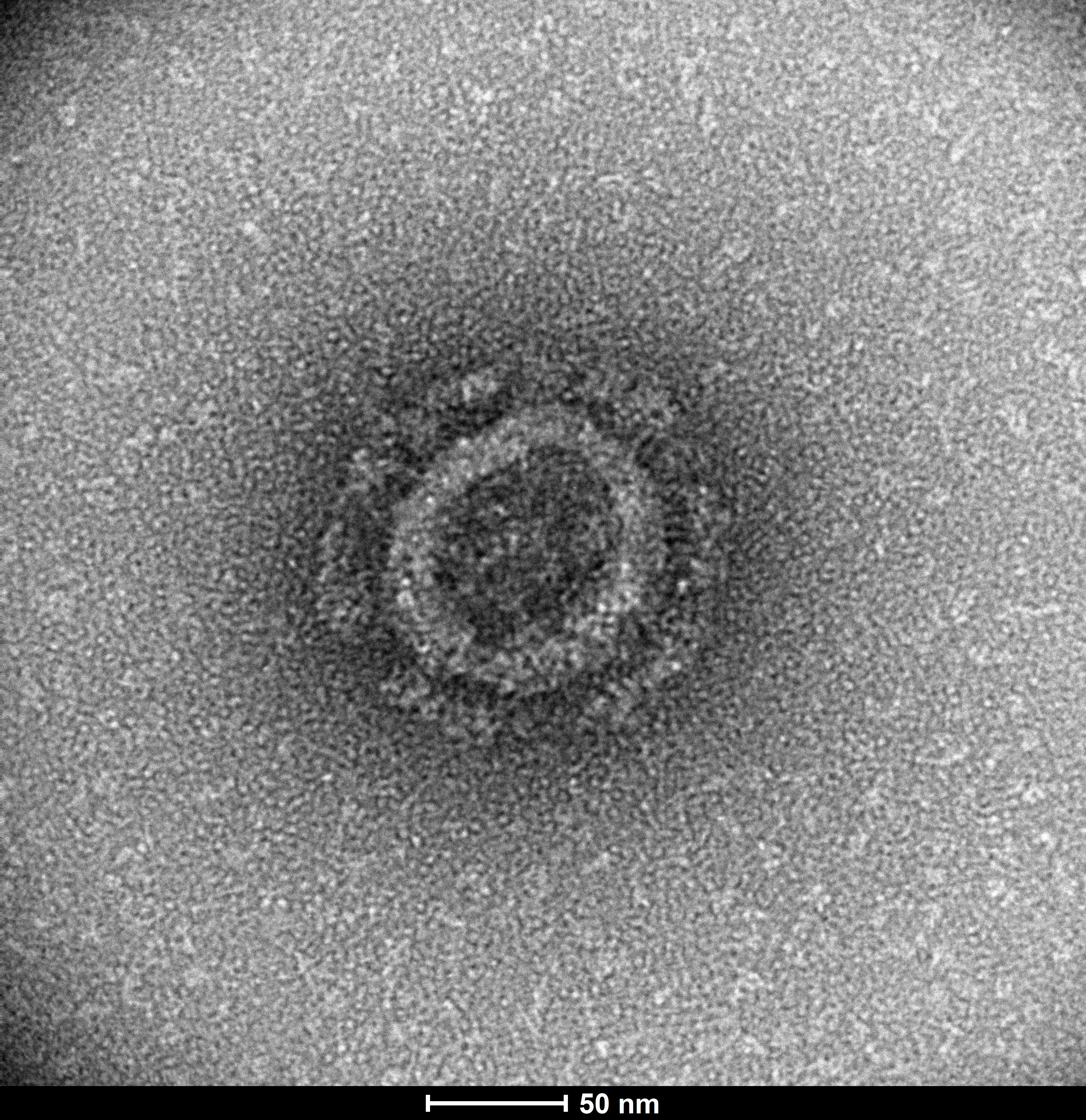

Supplement: Supplementary file 10 — Figures EV and Appendix Source Data [file 44318_2023_23_MOESM10_ESM.zip › Appendix Figure S8/S8A/17.jpg]

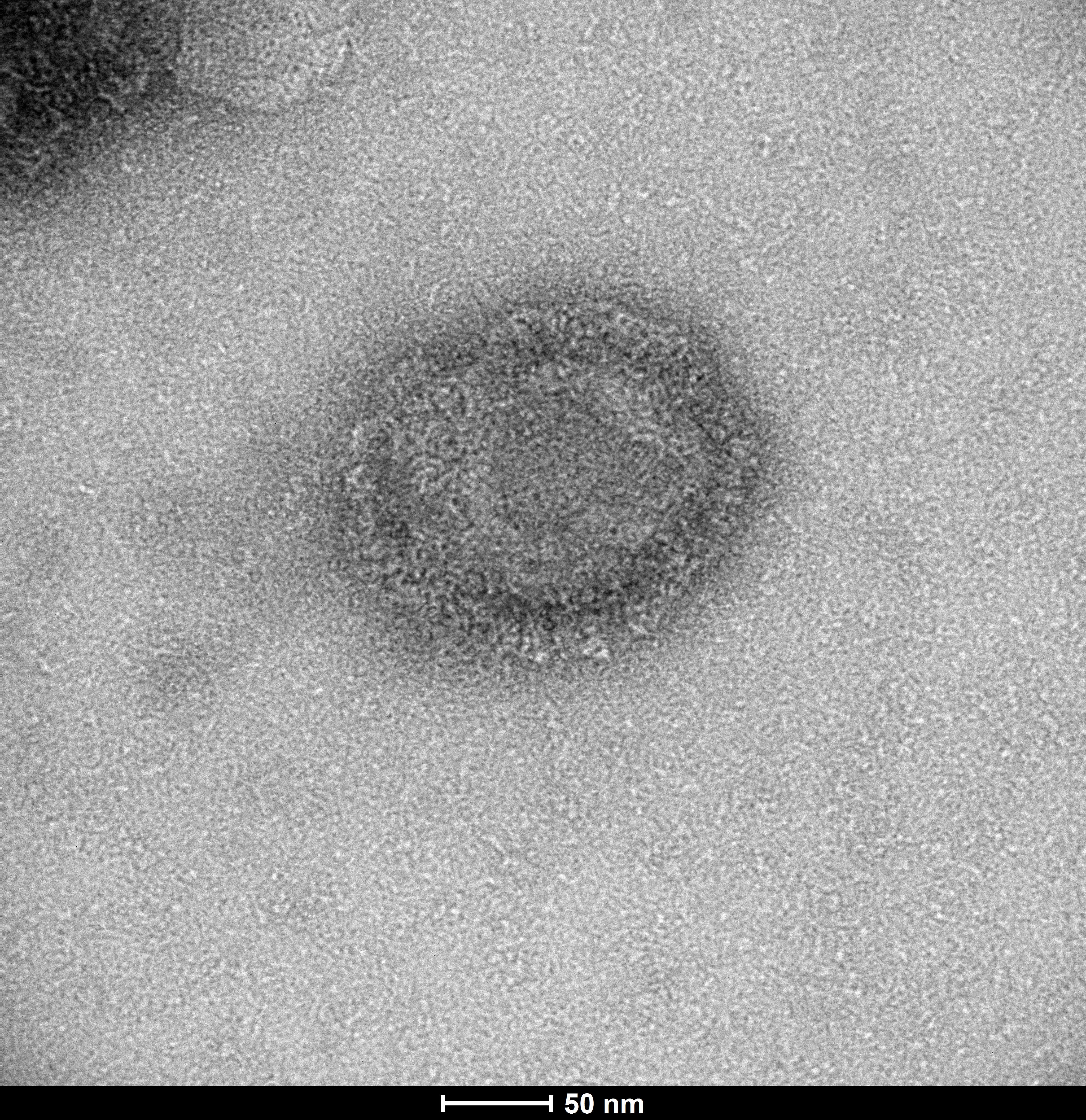

Supplement: Supplementary file 10 — Figures EV and Appendix Source Data [file 44318_2023_23_MOESM10_ESM.zip › Appendix Figure S8/S8A/19.jpg]

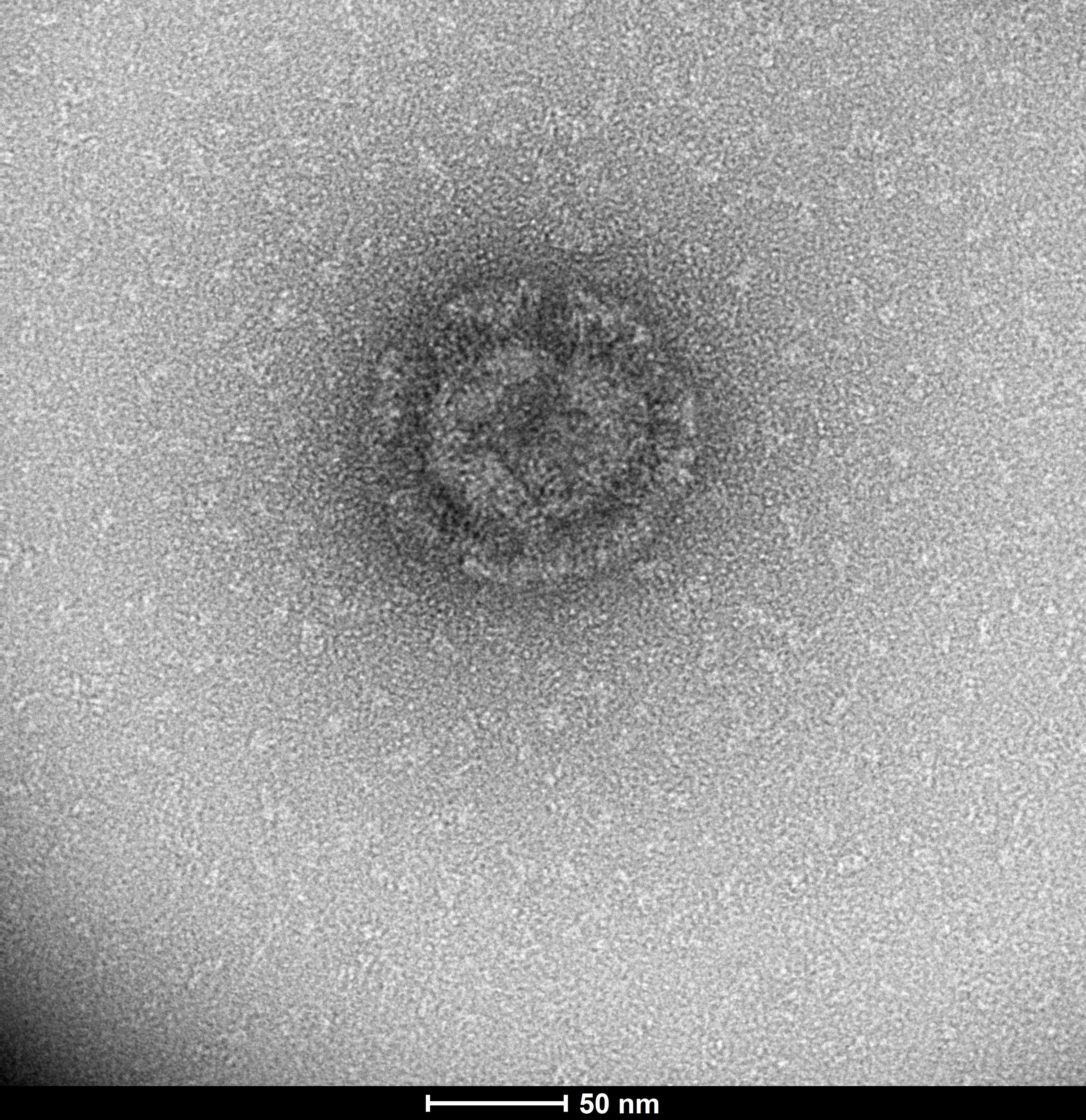

Supplement: Supplementary file 10 — Figures EV and Appendix Source Data [file 44318_2023_23_MOESM10_ESM.zip › Appendix Figure S8/S8A/21.jpg]

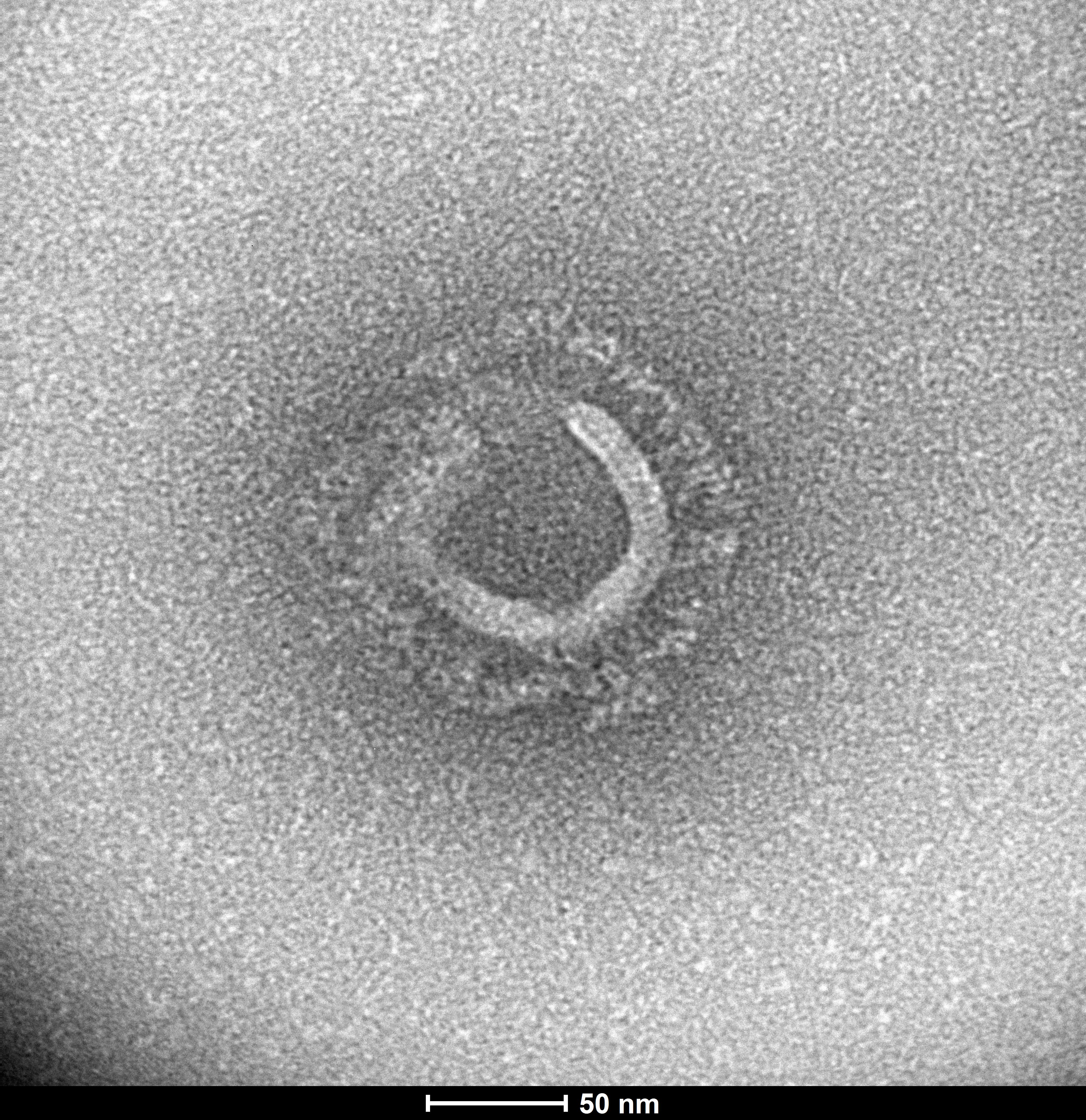

Supplement: Supplementary file 10 — Figures EV and Appendix Source Data [file 44318_2023_23_MOESM10_ESM.zip › Appendix Figure S8/S8A/25.jpg]

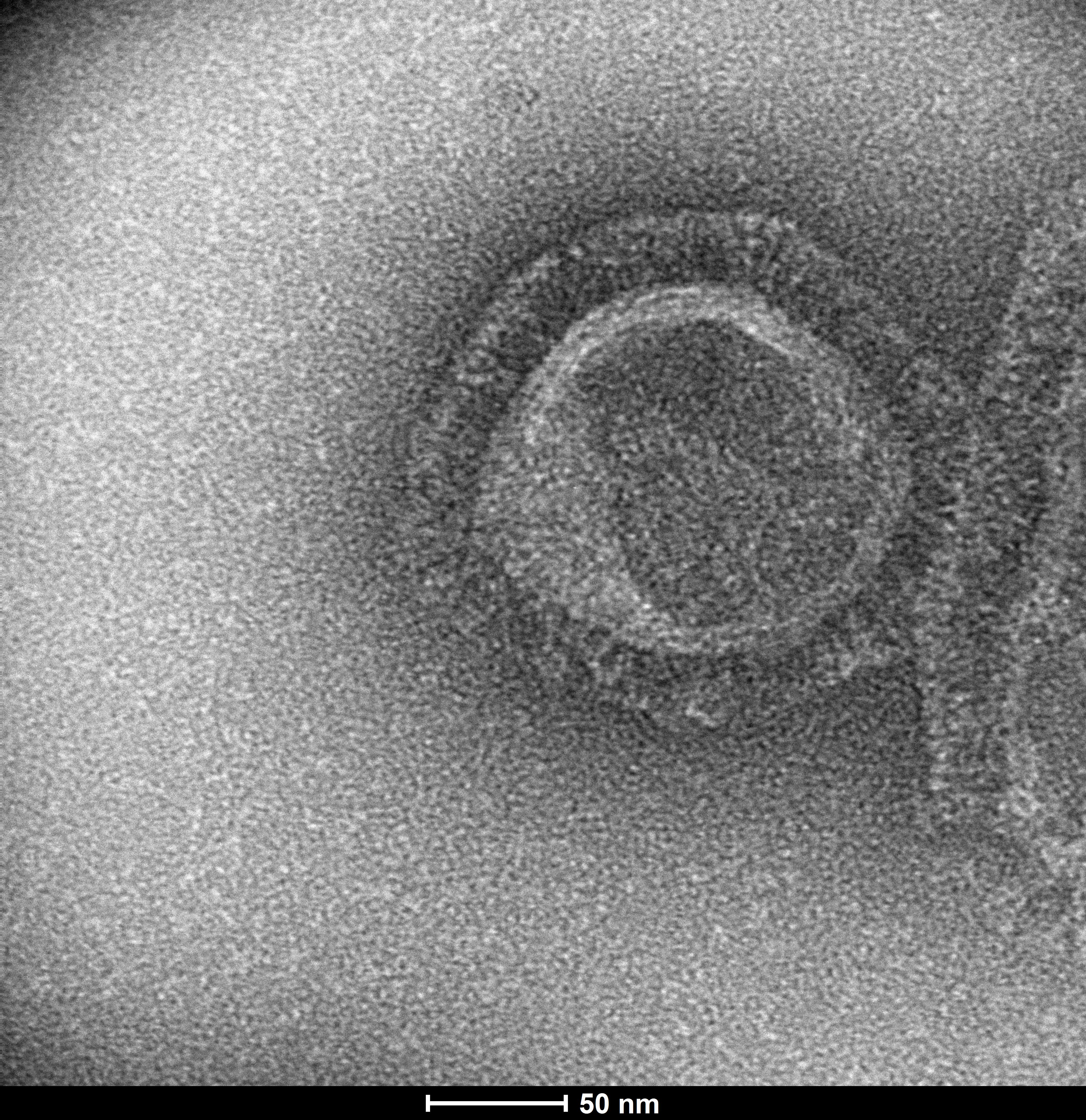

Supplement: Supplementary file 10 — Figures EV and Appendix Source Data [file 44318_2023_23_MOESM10_ESM.zip › Appendix Figure S8/S8A/28.jpg]

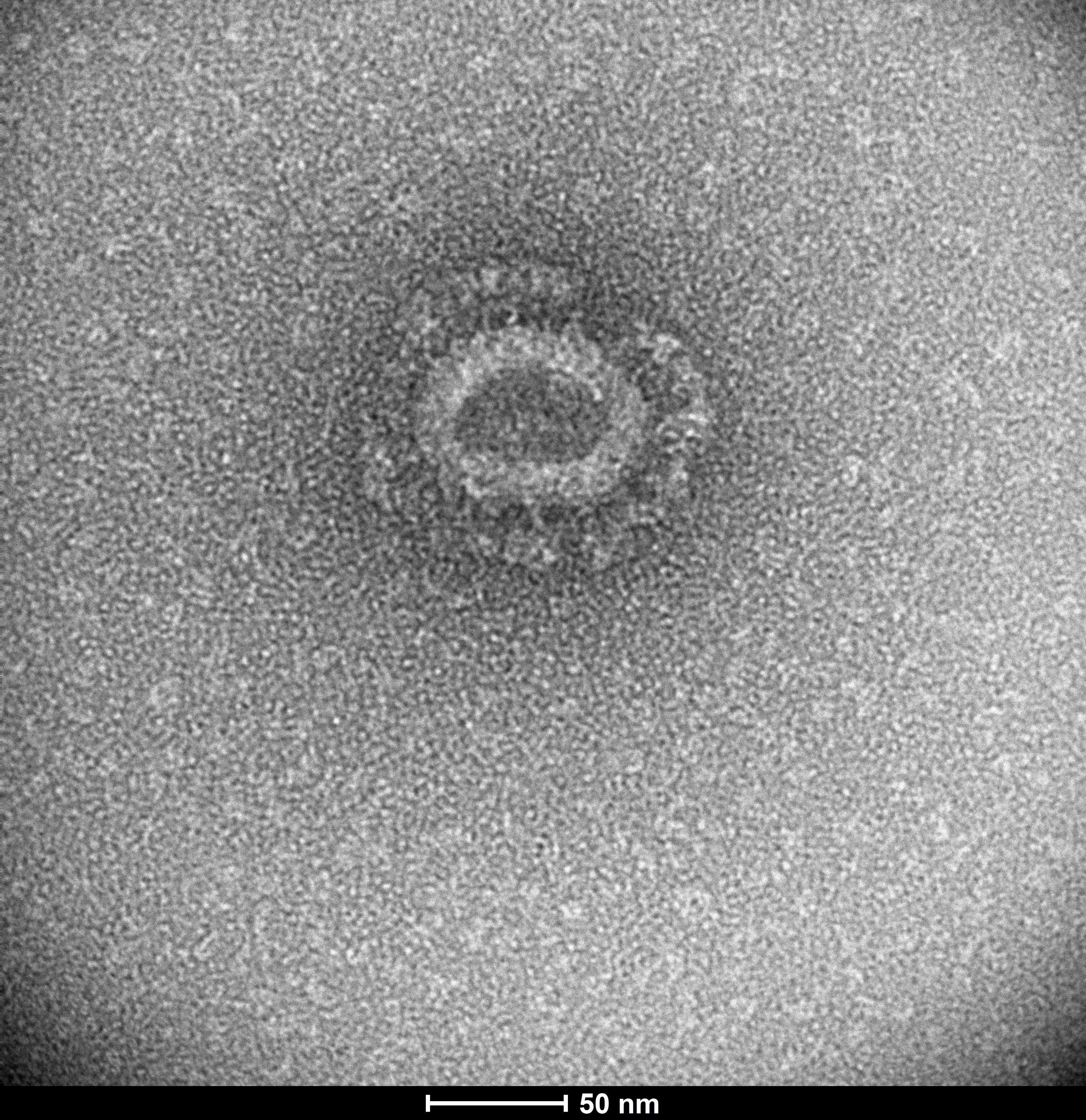

Supplement: Supplementary file 10 — Figures EV and Appendix Source Data [file 44318_2023_23_MOESM10_ESM.zip › Appendix Figure S8/S8A/6.jpg]

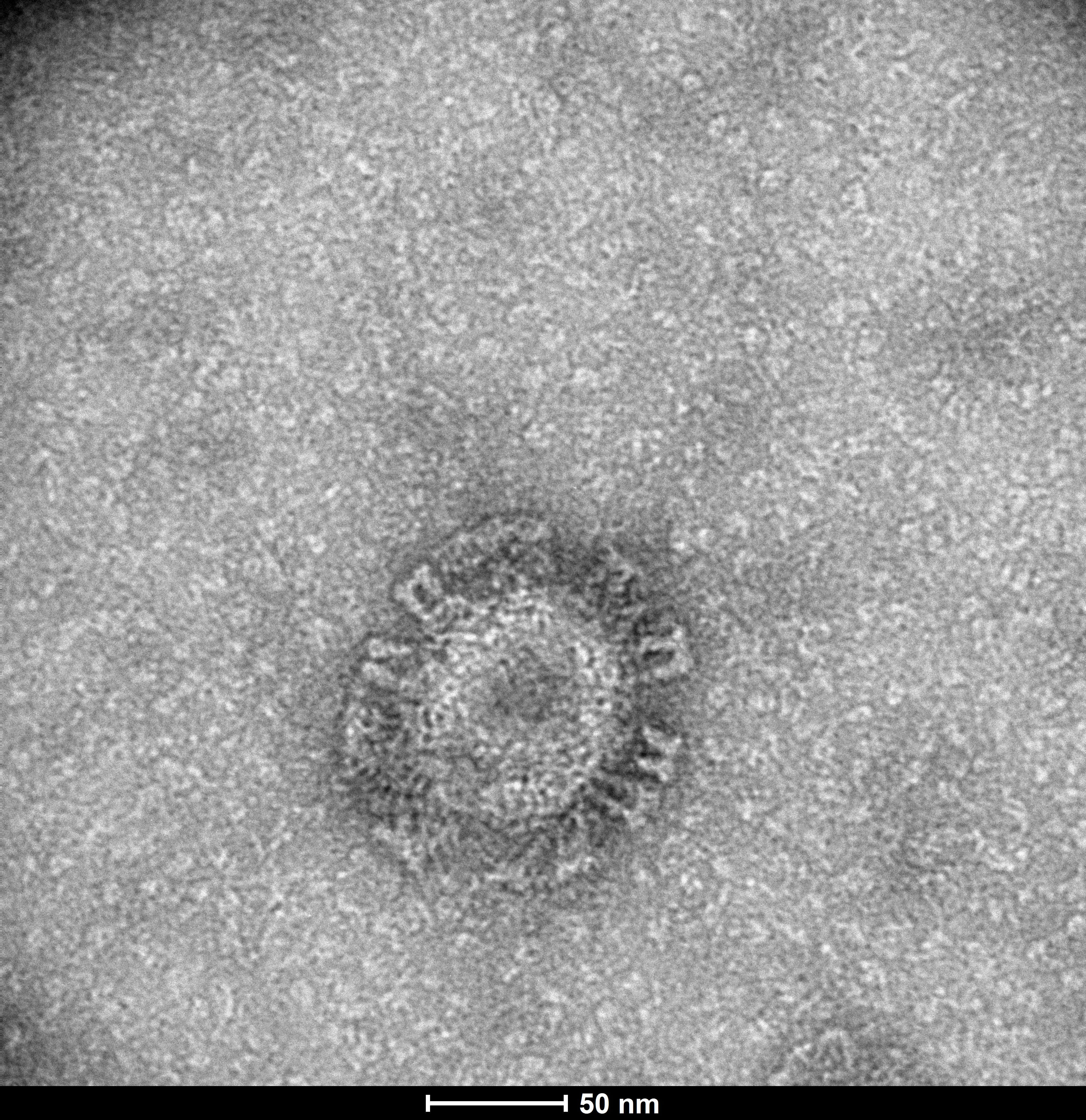

Supplement: Supplementary file 10 — Figures EV and Appendix Source Data [file 44318_2023_23_MOESM10_ESM.zip › Appendix Figure S8/S8B/156.jpg]

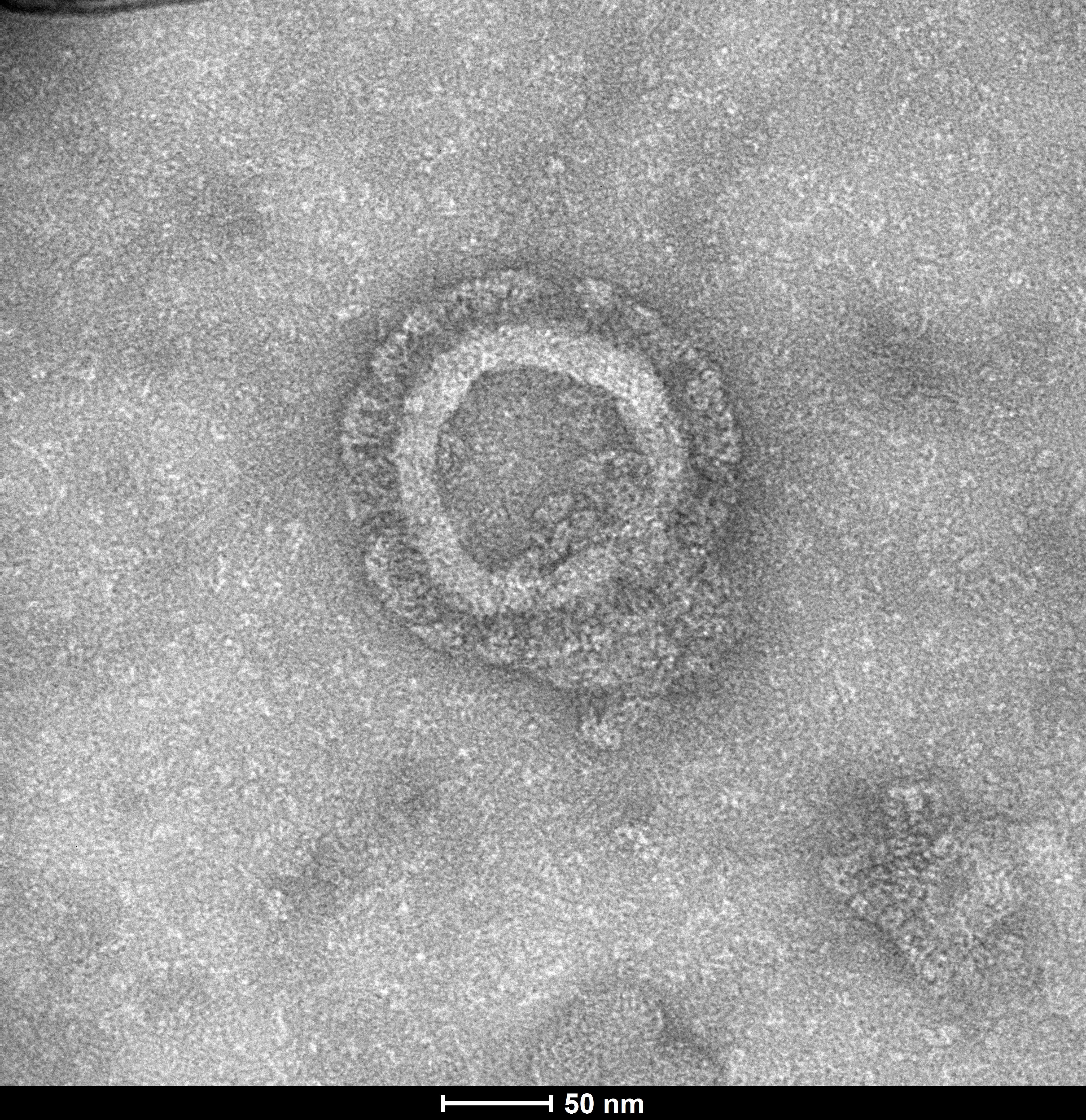

Supplement: Supplementary file 10 — Figures EV and Appendix Source Data [file 44318_2023_23_MOESM10_ESM.zip › Appendix Figure S8/S8B/173.jpg]

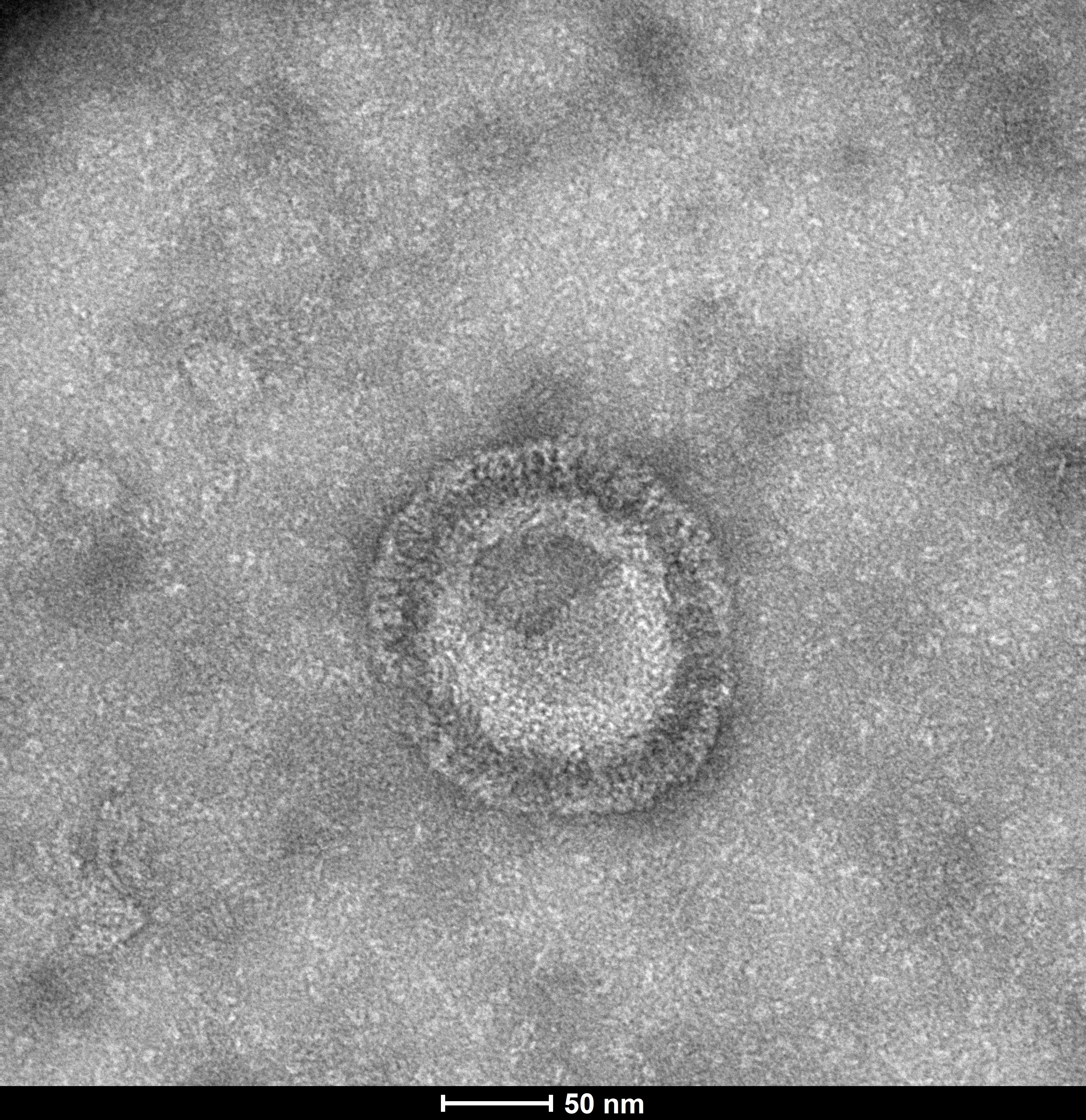

Supplement: Supplementary file 10 — Figures EV and Appendix Source Data [file 44318_2023_23_MOESM10_ESM.zip › Appendix Figure S8/S8B/176.jpg]

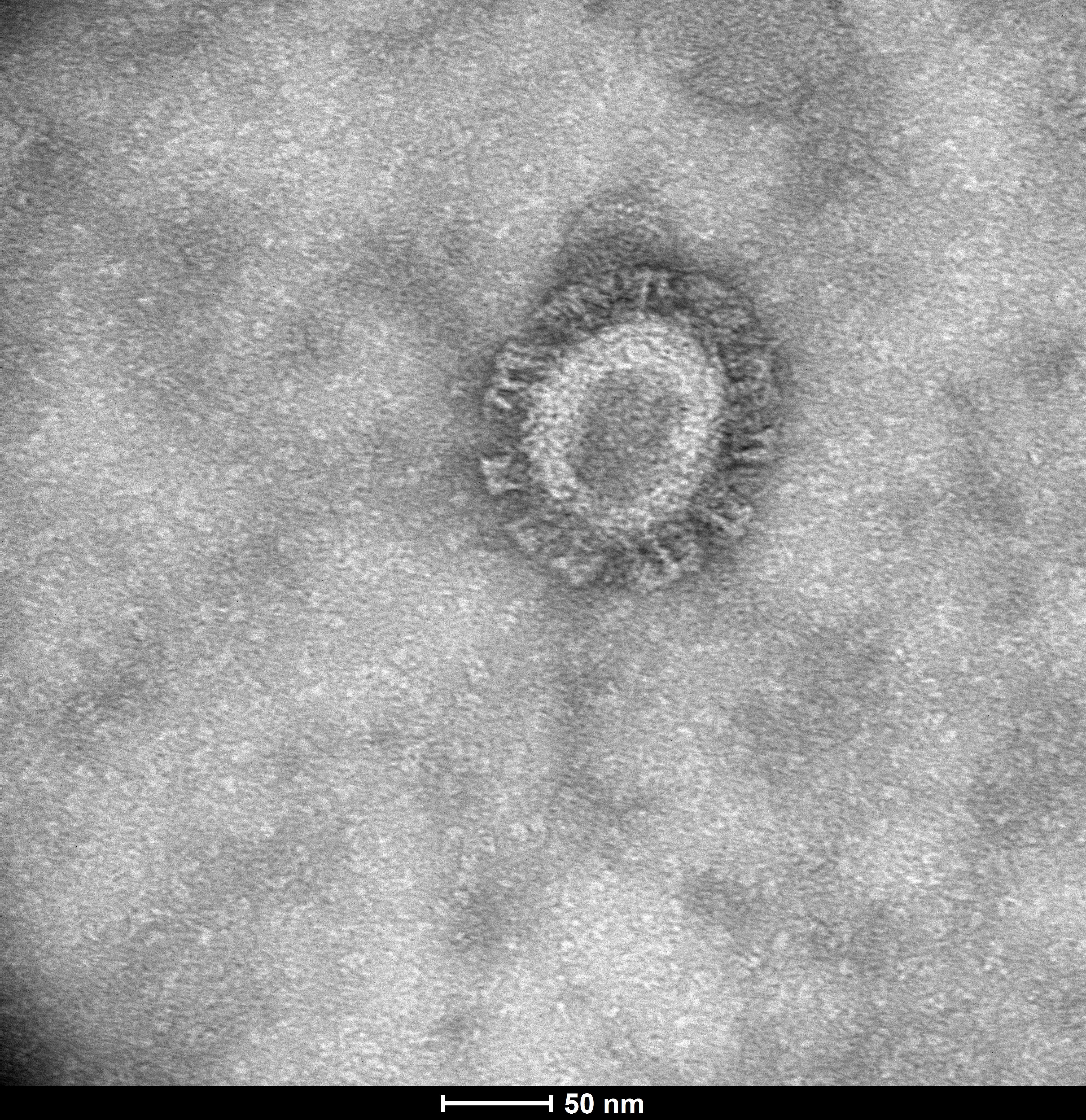

Supplement: Supplementary file 10 — Figures EV and Appendix Source Data [file 44318_2023_23_MOESM10_ESM.zip › Appendix Figure S8/S8B/190.jpg]

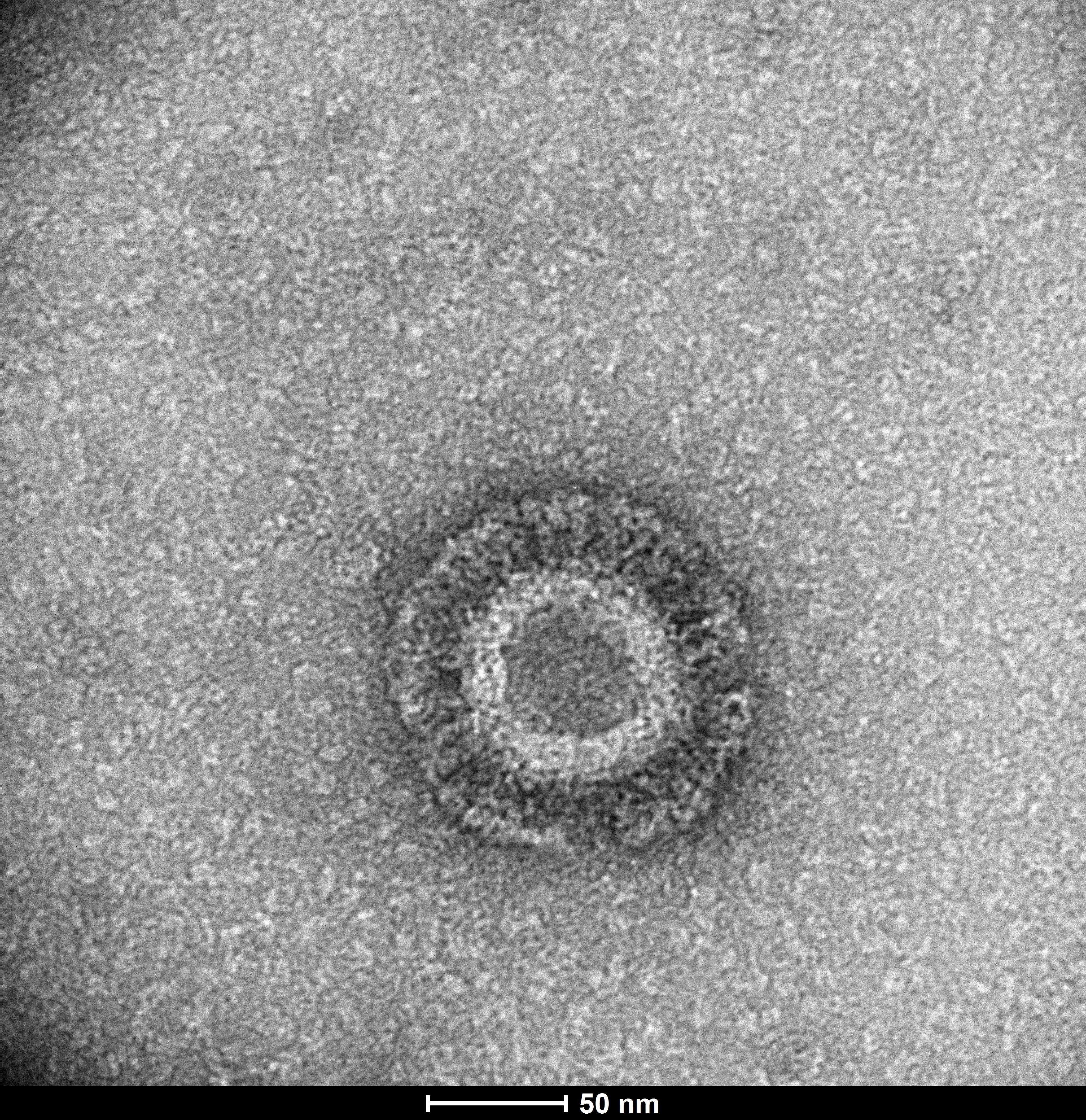

Supplement: Supplementary file 10 — Figures EV and Appendix Source Data [file 44318_2023_23_MOESM10_ESM.zip › Appendix Figure S8/S8B/20.jpg]

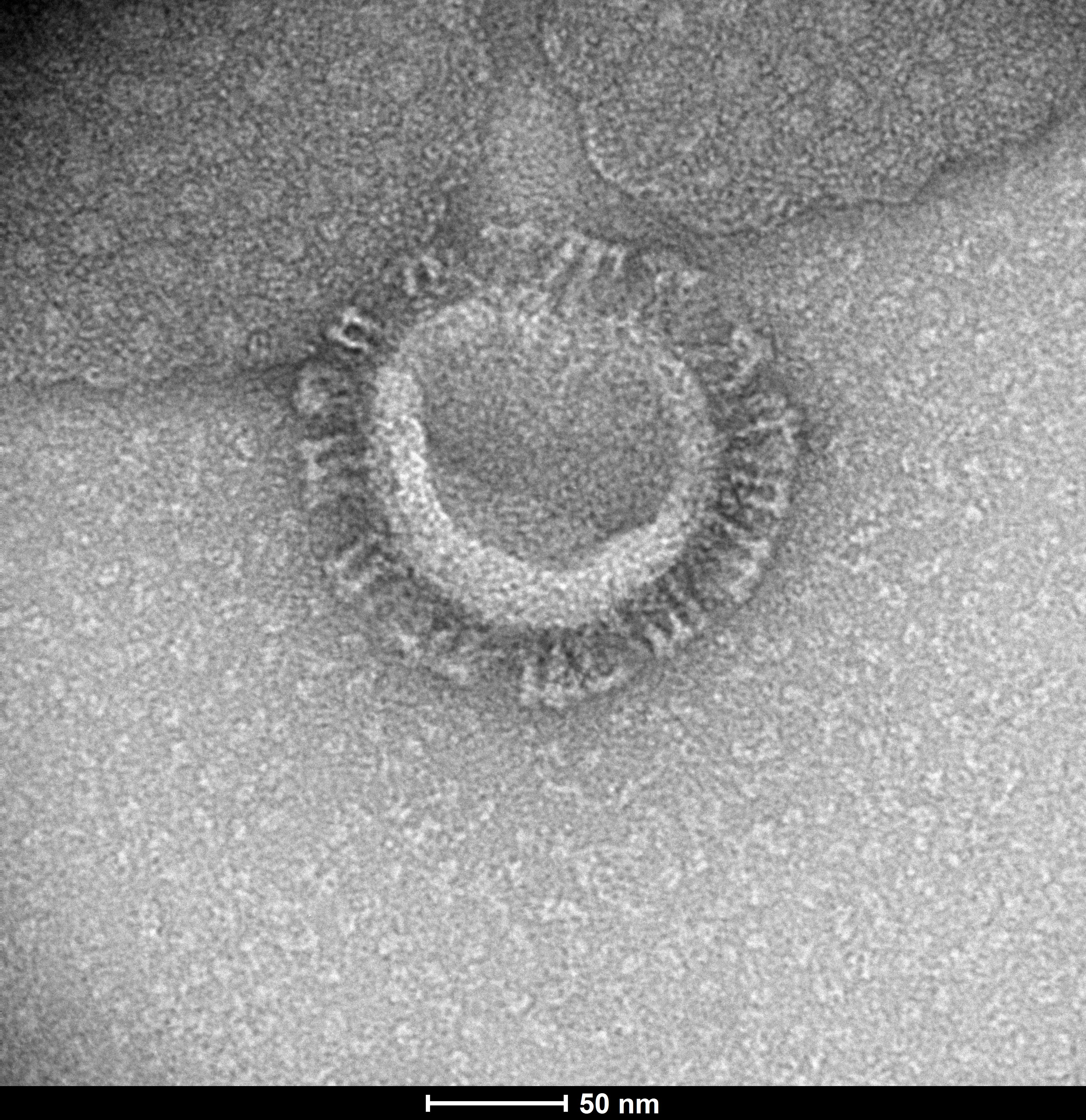

Supplement: Supplementary file 10 — Figures EV and Appendix Source Data [file 44318_2023_23_MOESM10_ESM.zip › Appendix Figure S8/S8B/65.jpg]

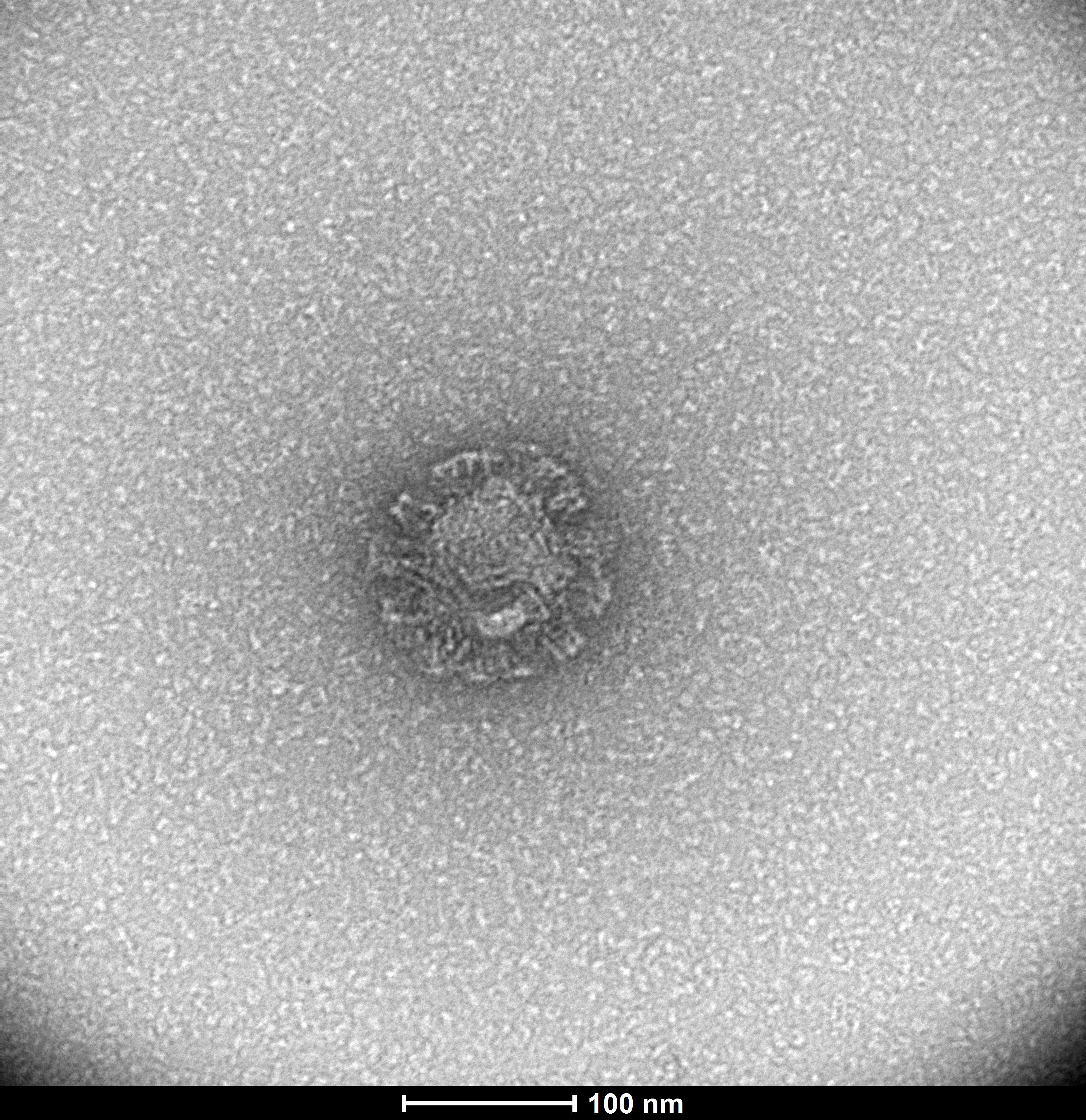

Supplement: Supplementary file 10 — Figures EV and Appendix Source Data [file 44318_2023_23_MOESM10_ESM.zip › Appendix Figure S8/S8B/71.jpg]

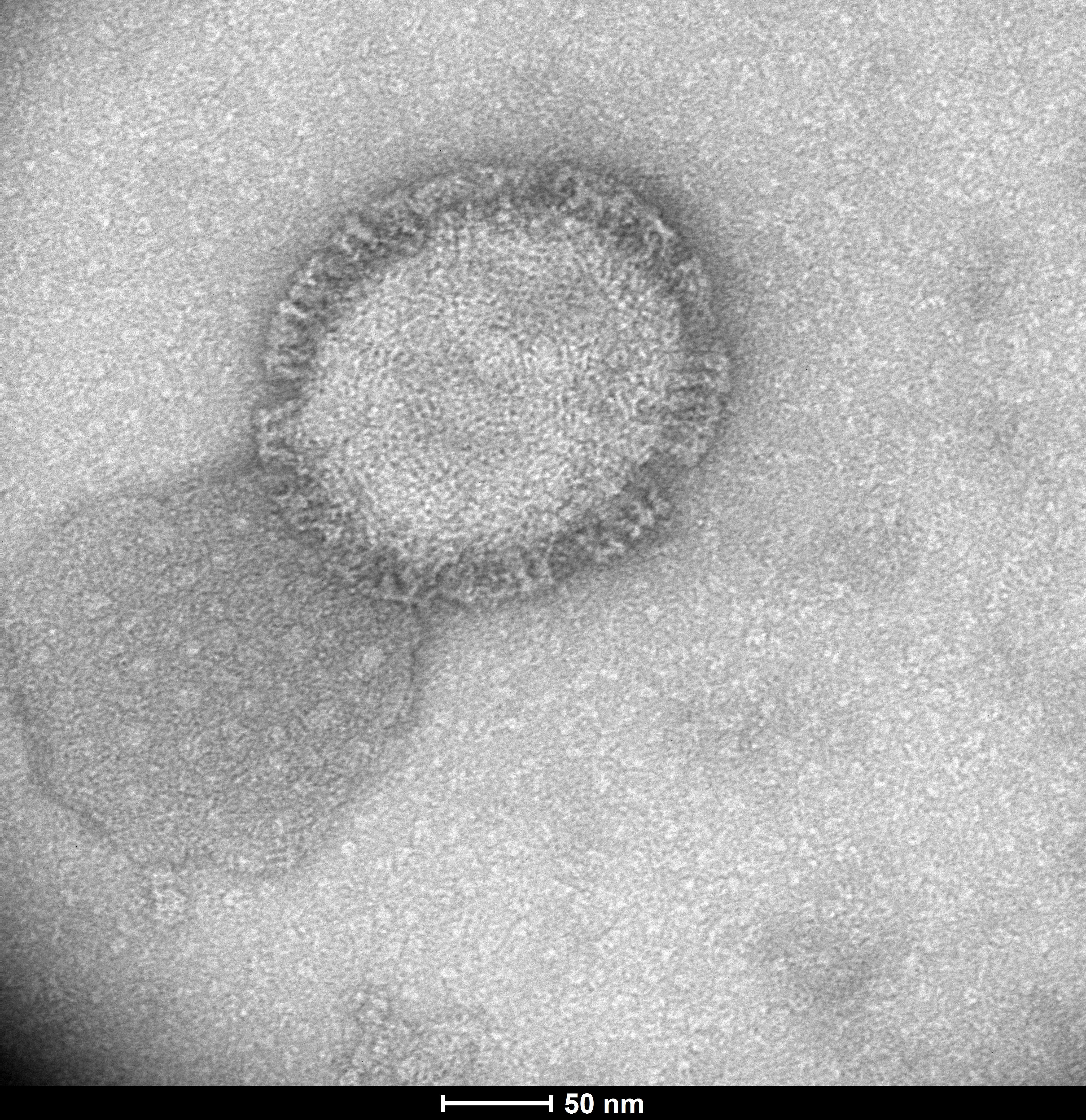

Supplement: Supplementary file 10 — Figures EV and Appendix Source Data [file 44318_2023_23_MOESM10_ESM.zip › Appendix Figure S8/S8B/91.jpg]

SourceData Fig. EV4A

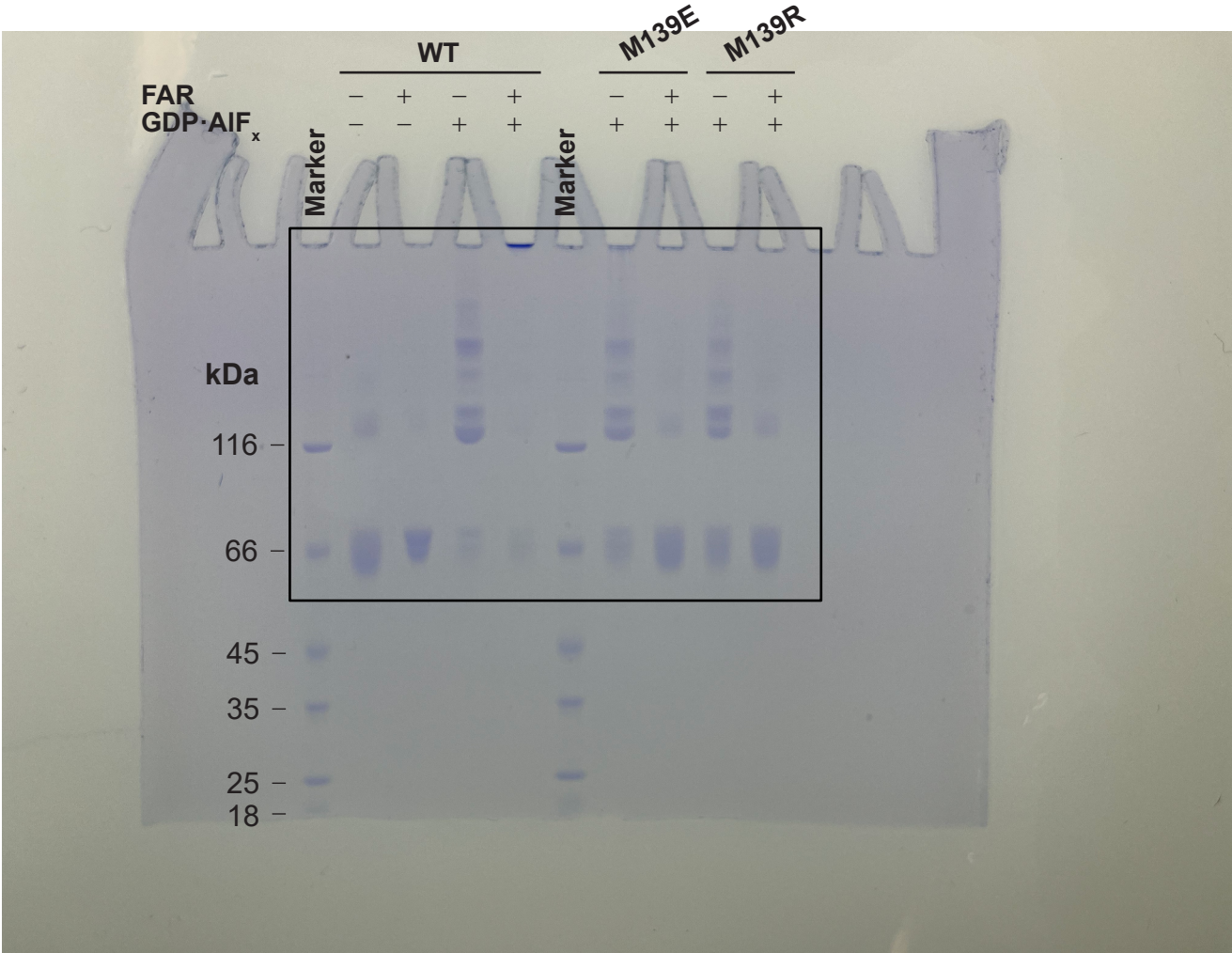

Supplement: Supplementary file 10 — Figures EV and Appendix Source Data [file 44318_2023_23_MOESM10_ESM.zip › Figure EV4/EV4A/SourceData_EV4A_crosslinking assay of M139 mutants.pdf]

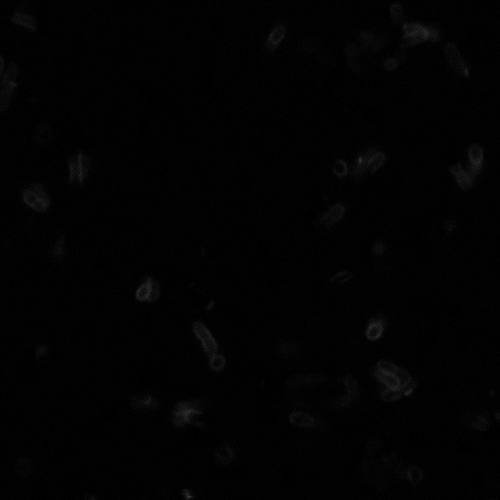

Supplement: Supplementary file 10 — Figures EV and Appendix Source Data [file 44318_2023_23_MOESM10_ESM.zip › Figure EV4/EV4C/M139E_crop.tif]

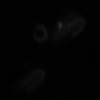

Supplement: Supplementary file 10 — Figures EV and Appendix Source Data [file 44318_2023_23_MOESM10_ESM.zip › Figure EV4/EV4C/M139E_detail.tif]

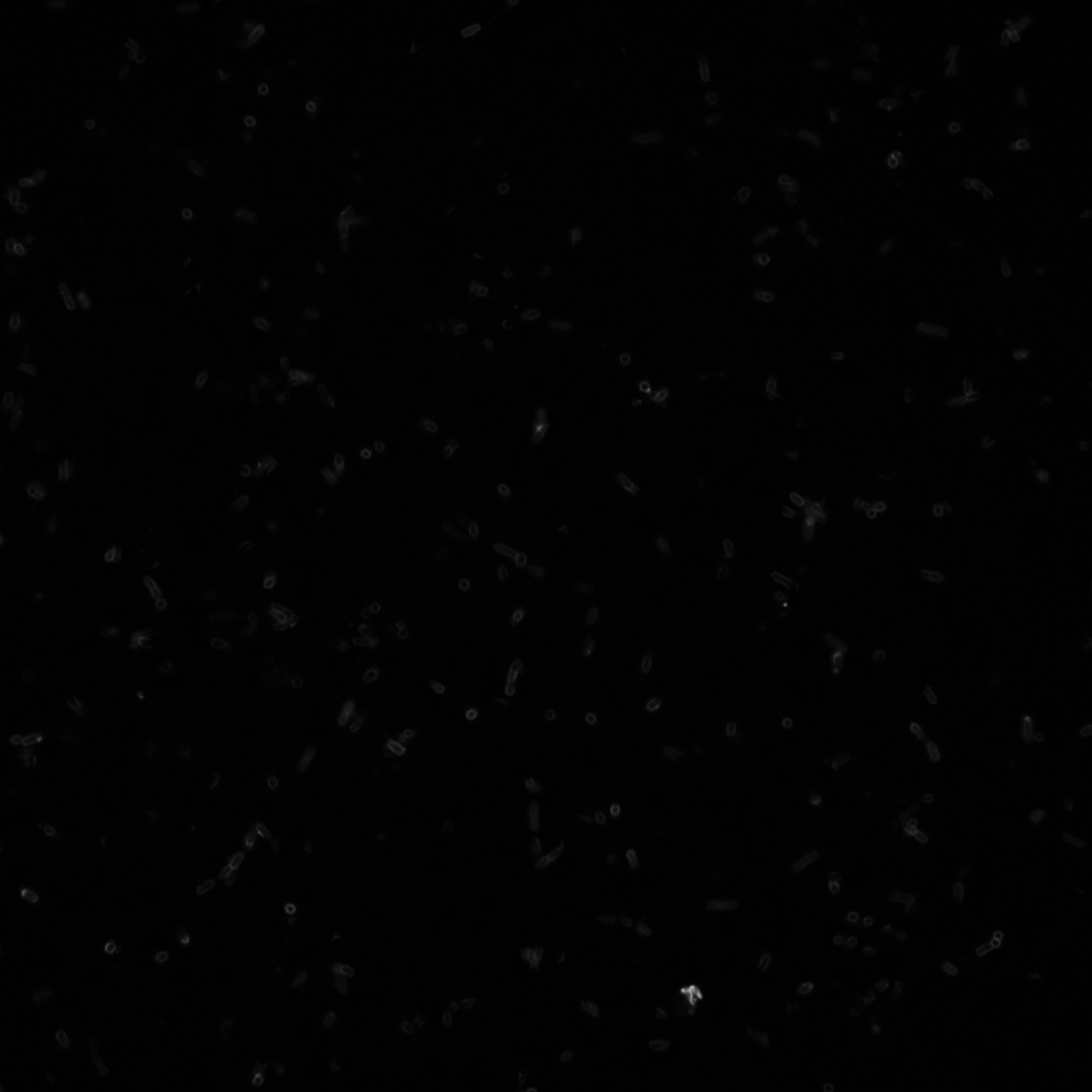

Supplement: Supplementary file 10 — Figures EV and Appendix Source Data [file 44318_2023_23_MOESM10_ESM.zip › Figure EV4/EV4C/M139E_original.tif]

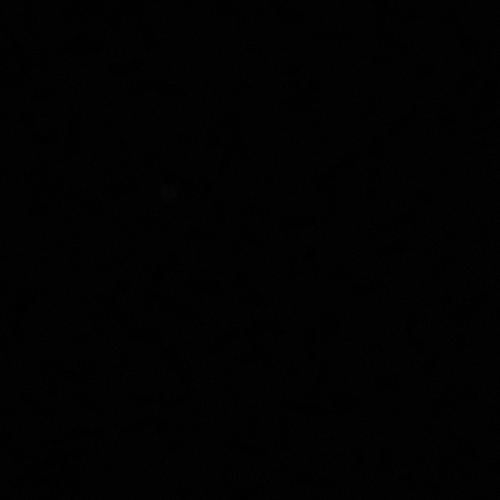

Supplement: Supplementary file 10 — Figures EV and Appendix Source Data [file 44318_2023_23_MOESM10_ESM.zip › Figure EV4/EV4C/M139R_crop.tif]

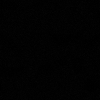

Supplement: Supplementary file 10 — Figures EV and Appendix Source Data [file 44318_2023_23_MOESM10_ESM.zip › Figure EV4/EV4C/M139R_detail.tif]

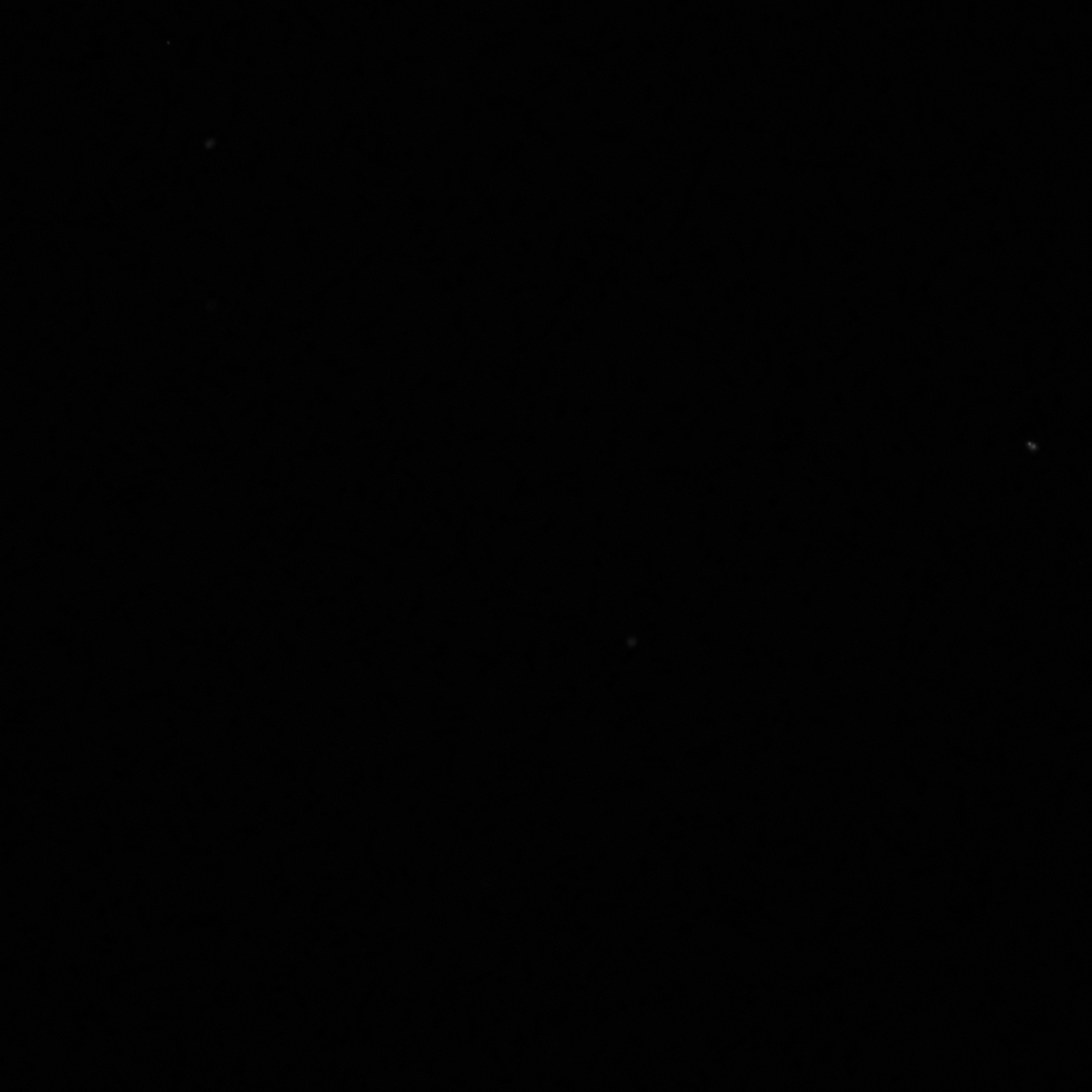

Supplement: Supplementary file 10 — Figures EV and Appendix Source Data [file 44318_2023_23_MOESM10_ESM.zip › Figure EV4/EV4C/M139R_original.tif]

SourceData Fig. EV4E

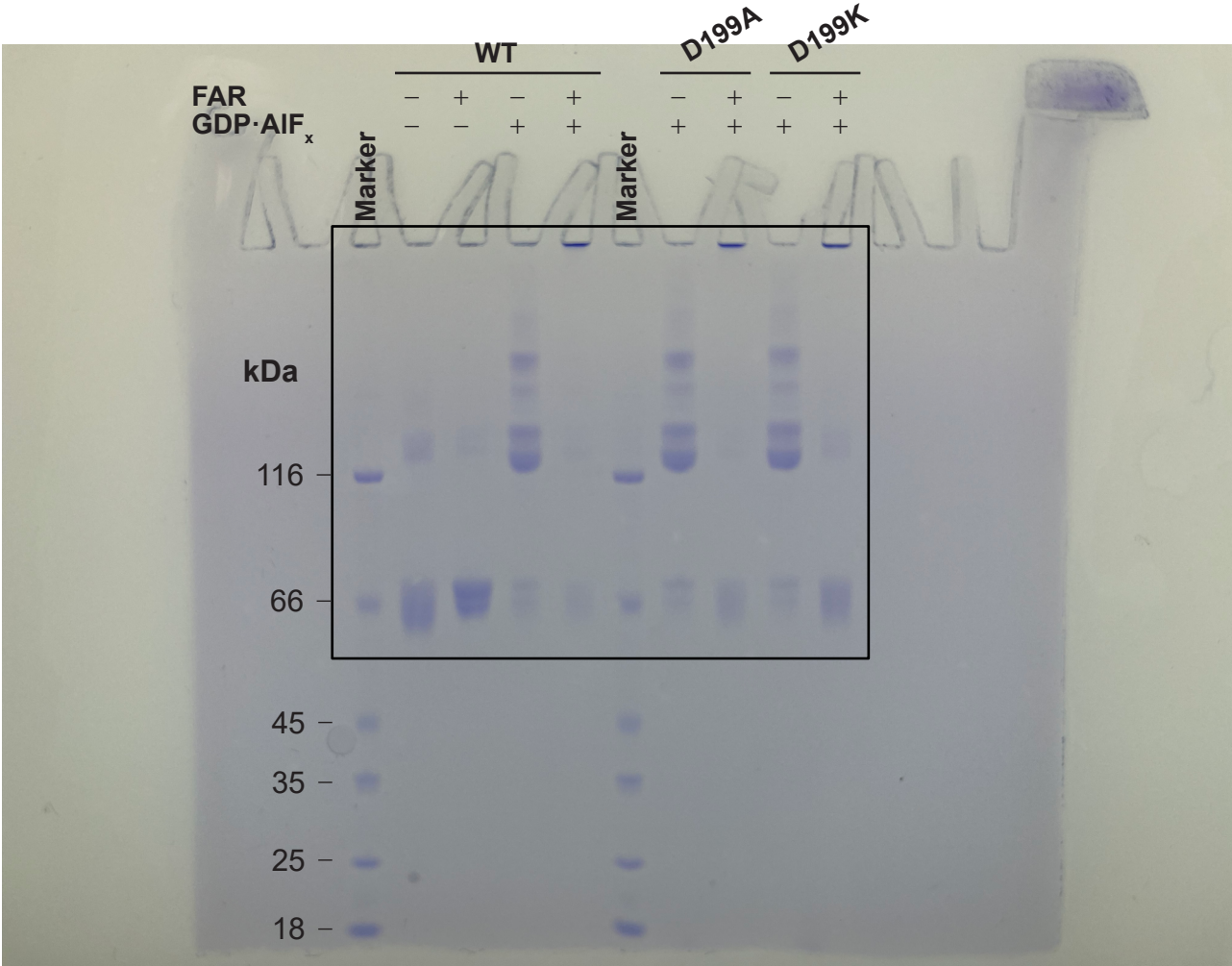

Supplement: Supplementary file 10 — Figures EV and Appendix Source Data [file 44318_2023_23_MOESM10_ESM.zip › Figure EV4/EV4E/SourceData_EV4E_crosslinking assay of D199 mutants.pdf]

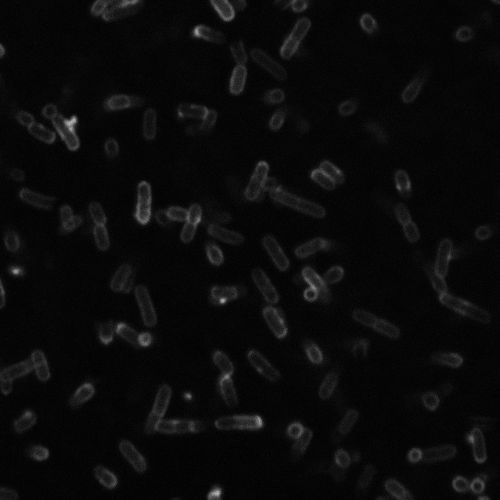

Supplement: Supplementary file 10 — Figures EV and Appendix Source Data [file 44318_2023_23_MOESM10_ESM.zip › Figure EV4/EV4F/D199A_crop.tif]

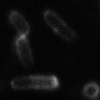

Supplement: Supplementary file 10 — Figures EV and Appendix Source Data [file 44318_2023_23_MOESM10_ESM.zip › Figure EV4/EV4F/D199A_detail.tif]

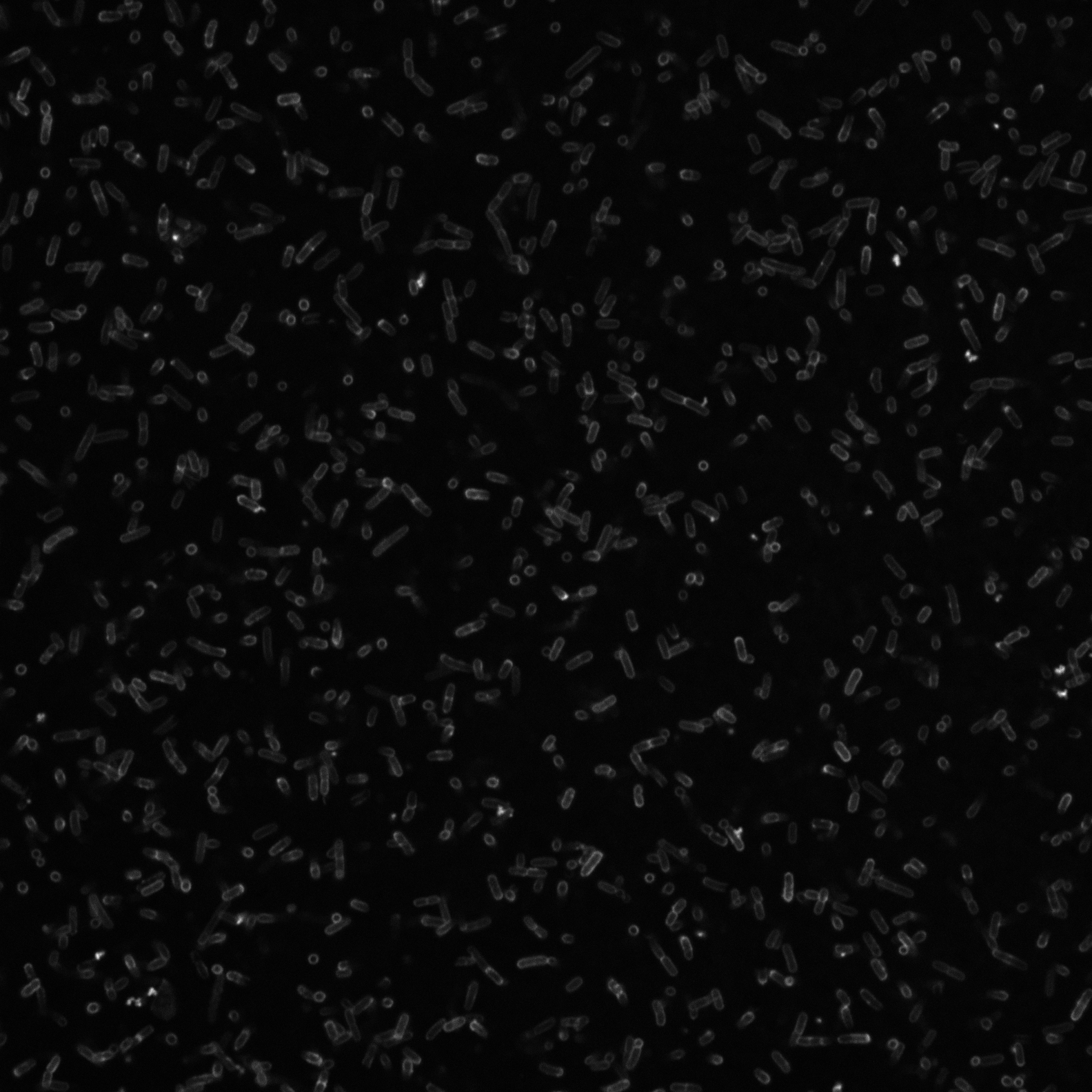

Supplement: Supplementary file 10 — Figures EV and Appendix Source Data [file 44318_2023_23_MOESM10_ESM.zip › Figure EV4/EV4F/D199A_original.tif]

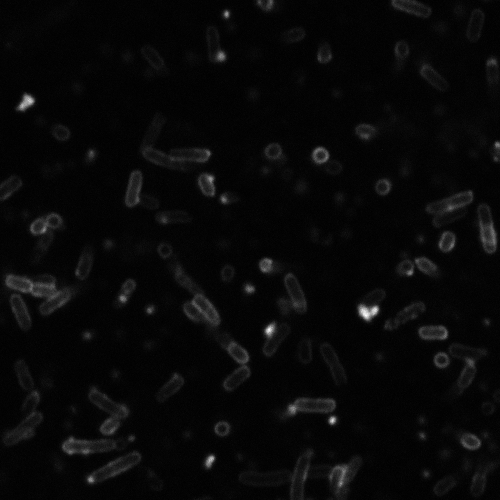

Supplement: Supplementary file 10 — Figures EV and Appendix Source Data [file 44318_2023_23_MOESM10_ESM.zip › Figure EV4/EV4F/D199K_crop.tif]

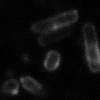

Supplement: Supplementary file 10 — Figures EV and Appendix Source Data [file 44318_2023_23_MOESM10_ESM.zip › Figure EV4/EV4F/D199K_detail.tif]

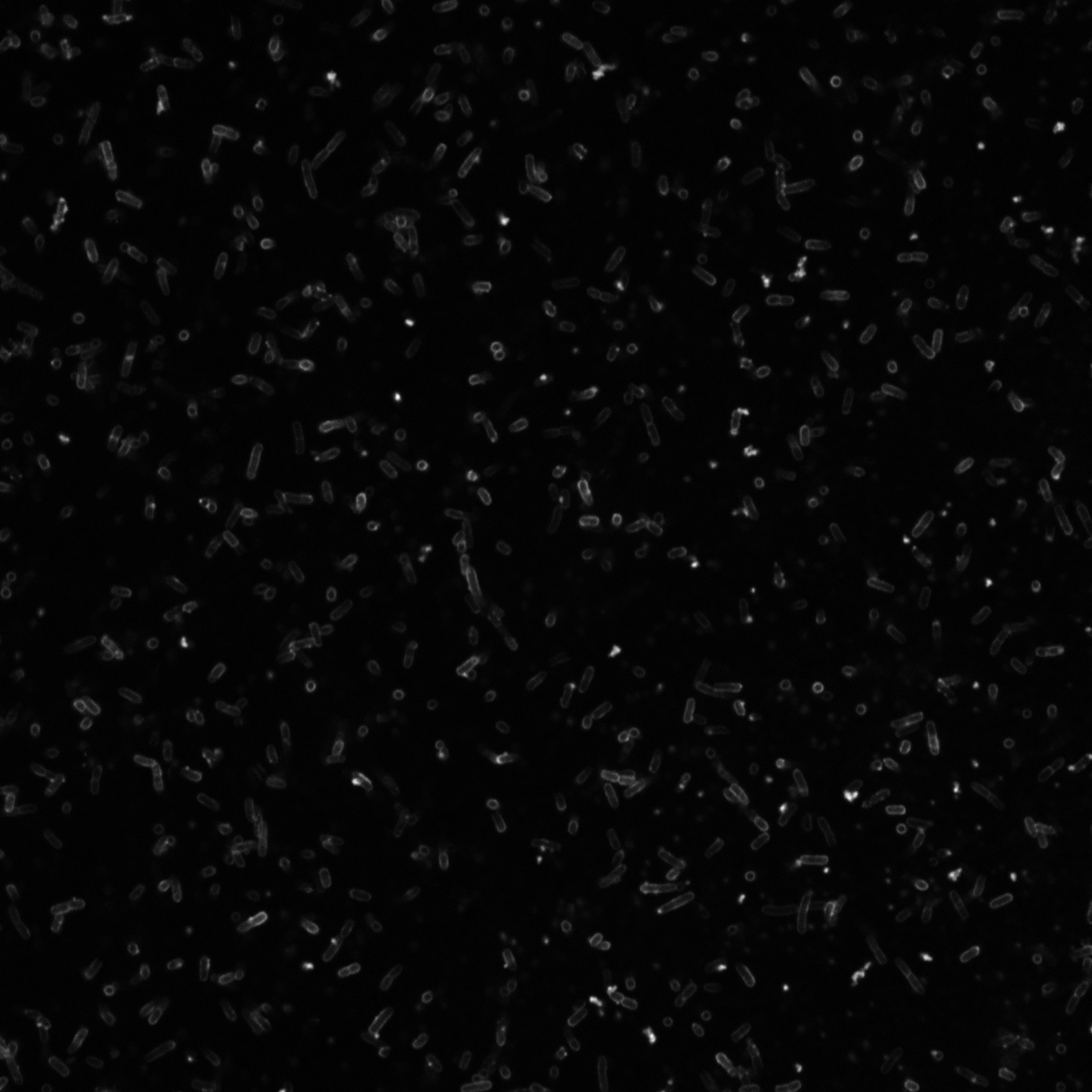

Supplement: Supplementary file 10 — Figures EV and Appendix Source Data [file 44318_2023_23_MOESM10_ESM.zip › Figure EV4/EV4F/D199K_original.tif]
