# Supplementary material for: Structural insights into the activation mechanism of antimicrobial GBP1
Source: EMBO J. 2024 Jan 24;43(4):615–36. doi: 10.1038/s44318-023-00023-y (PMC10897159; doi:10.1038/s44318-023-00023-y)
Supplement: Supplementary file 12 — Expanded View Figures [file 44318_2023_23_MOESM12_ESM.pdf]

Expanded View Figures

Figure EV1. Subtomogram averaging pipeline of membrane-bound GBP1.

(**A**) Cropped segments from GBP1 tomograms are displayed by "dynamo_gallery." (**B**) Two steps of 3D classification were implemented in pixel size of 17.6 Å and 4.4 Å. Boxes of the dashed line denote the selected classes for next steps. (**C**) 3D refinement result of the selected class and gold-standard FSC curve. Map was low-passed to 25 Å.

© The Author(s)

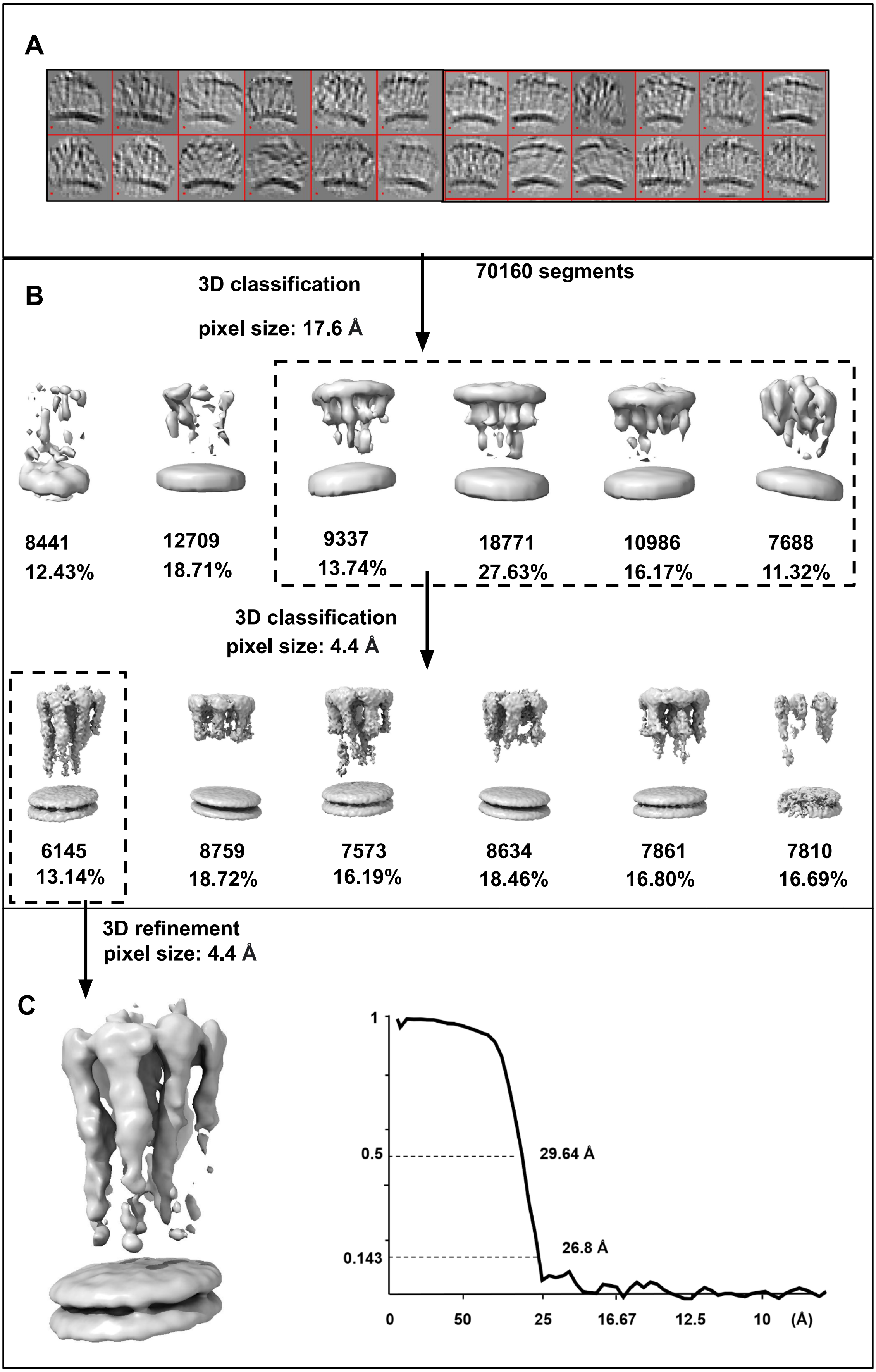
A
B
3D classification
pixel size: 17.6 Å
70160 segments
8441
12.43%
12709
18.71%
9337
13.74%
18771
27.63%
10986
16.17%
7688
11.32%
3D classification
pixel size: 4.4 Å
6145
13.14%
8759
18.72%
7573
16.19%
8634
18.46%
7861
16.80%
7810
16.69%
3D refinement
pixel size: 4.4 Å
C
1
0.5
0.143
29.64 Å
26.8 Å
0
50
25
16.67
12.5
10
(Å)

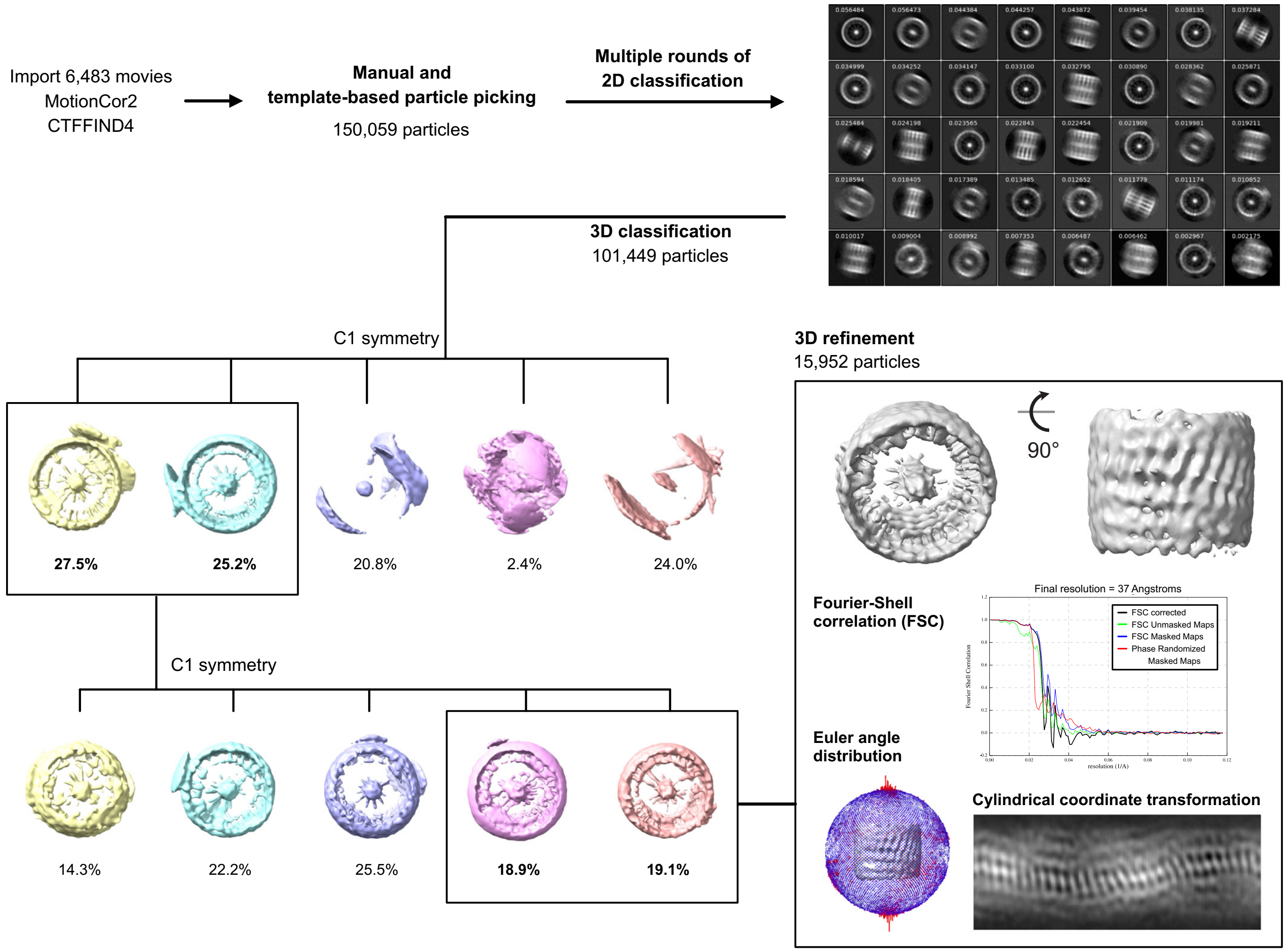

Figure EV2. Image processing workflow for polymeric GBP1 disks.

Boxes denote selected 3D classes for next steps. 3D refinement result is shown together with gold-standard FSC curves, Euler angle distribution of particles contributing to the final reconstruction, and cylindrical coordinate transformation visualizing the surface of the 3D reconstruction at the heights of the peripheral LG domains.

 © The Author(s)

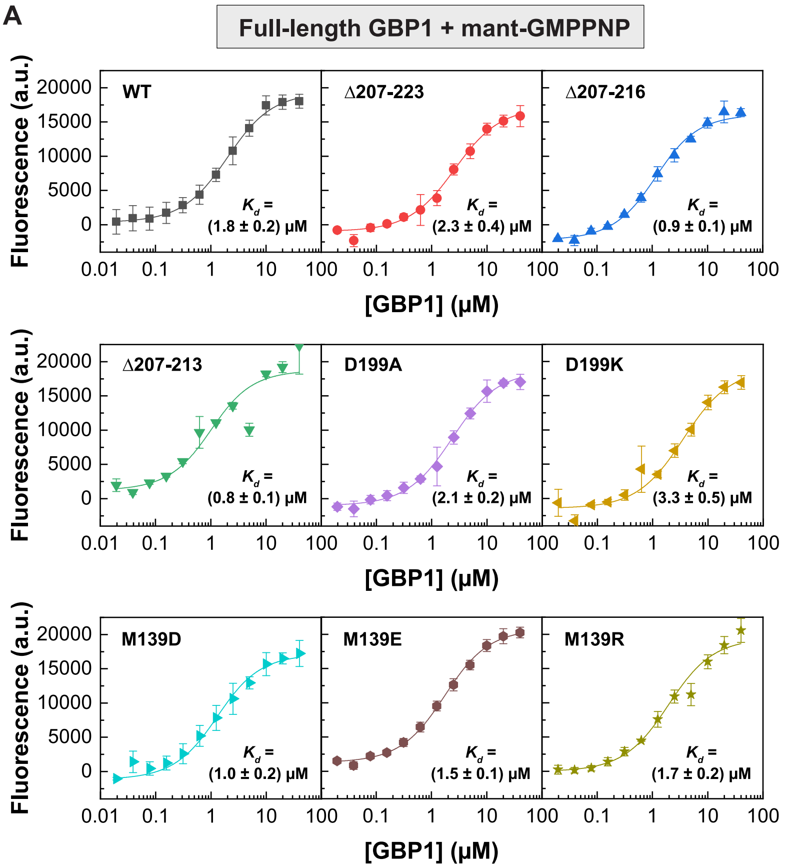

Figure EV3. Nucleotide binding.

(**A**) Fluorescence of mant-GMPPNP (0.5 μM) at varying GBP1 concentration for indicated full-length constructs. Data points are averages from three independent experiments and are represented by mean ± SD. Equilibrium dissociation constants K_d were calculated by fitting a quadratic equation to data using Eq. 1. (**B**) Fluorescence of mant-GDP (0.5 μM) at varying GBP1 concentration for indicated isolated LG domain constructs. Data points are averages from three independent experiments and are represented by mean ± SD. Equilibrium dissociation constants K_d were calculated by fitting a quadratic equation to data using Eq. 1. Source data are available online for this figure.

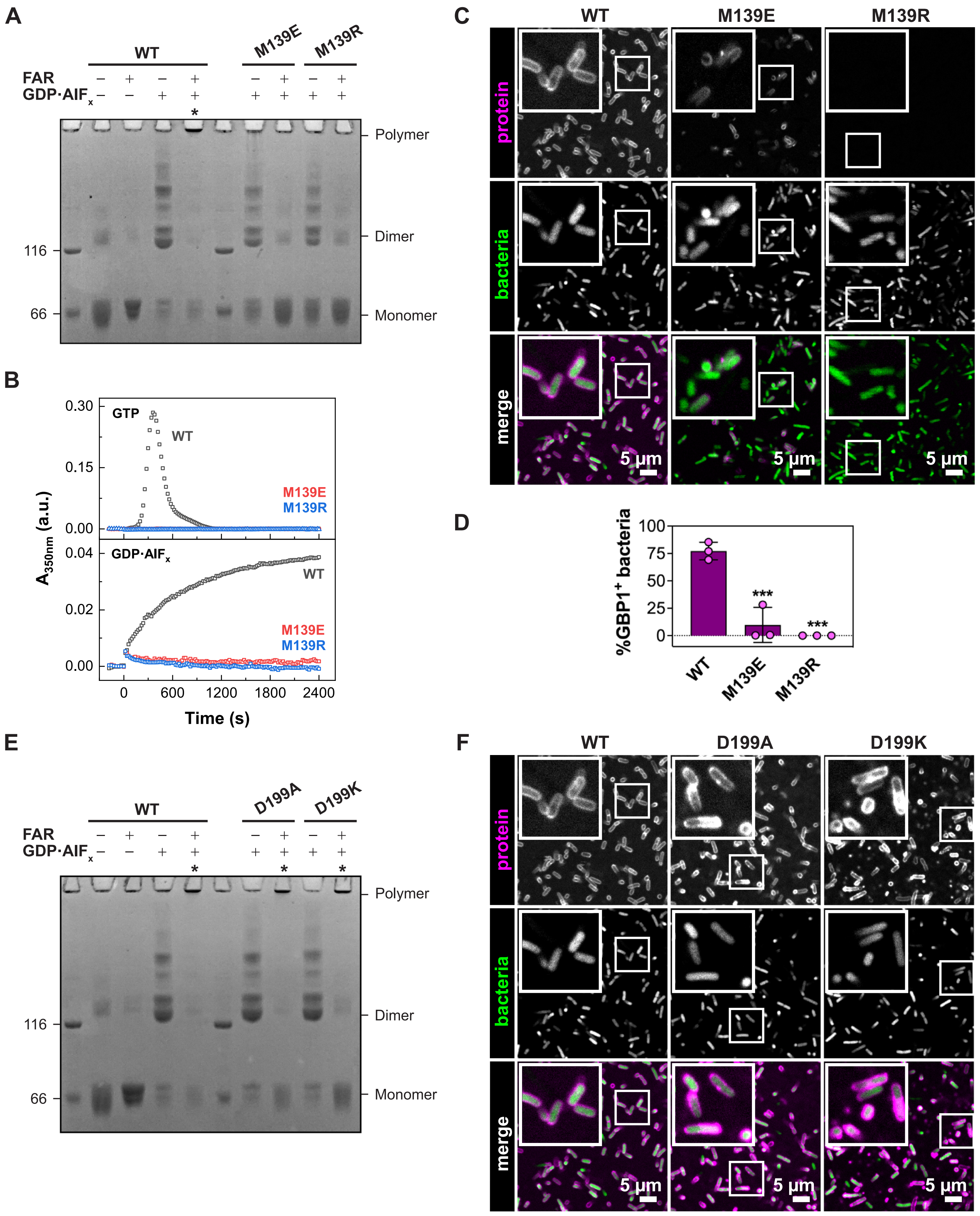
A
WT
M139E
M139R
FAR
GDP·AlF$_x$
*
Polymer
Dimer
Monomer
116
66
B
GTP
WT
M139E
M139R
GDP·AlF$_x$
A_{350nm} (a.u.)
0.30
0.15
0.00
0.04
0.02
0
600
1200
1800
2400
Time (s)
C
protein
bacteria
merge
5 µm
D
%GBP1$^+$ bacteria
100
75
50
25
***
E
D199A
D199K
F

 © The Author(s)

Figure EV4. Oligomerization of dimerization-capable M139 and pivot point mutants.

(**A**) Crosslinking assay of M139 mutants. The respective oligomeric states based on molecular weight are indicated. Asterisks indicate polymerization. FAR farnesylated. (**B**) Light scattering-based polymerization assay of M139 mutants. Polymerization is induced by GTP (top) and GDP•AlF_x (bottom). WT data is the same as shown in Fig. 2D and provided here for comparison. (**C**) Confocal microscopy images of wild-type GBP1 and M139 mutants targeting pathogenic *E. coli*, as described in the legend of Fig. 3F. (**D**) Quantification of GBP1-targeted bacteria in in vitro binding assay, as described in the legend of Fig. 3G. (**E**) Cross-linking assay of D199 mutants. The respective oligomeric states based on molecular weight are indicated. Asterisks indicate successful polymerization. FAR farnesylated. (**F**) Confocal microscopy images of wild-type GBP1 and D199 mutants targeting pathogenic *E. coli*, as described in Fig. 3F. Source data are available online for this figure.

© The Author(s)

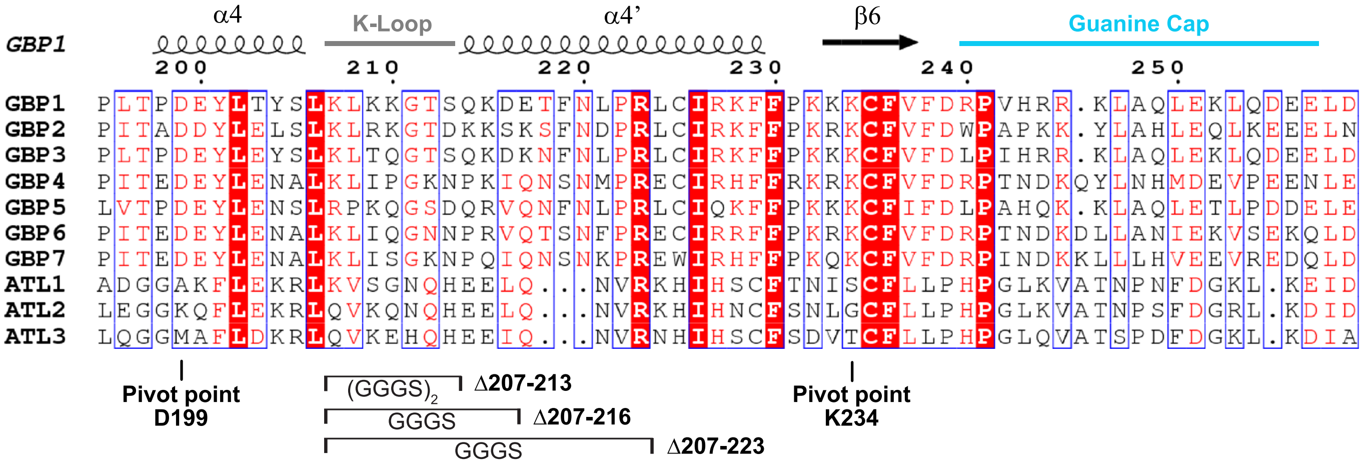

Figure EV5. Sequence alignment of the intramolecular lever.

Structural elements of PDB 1dg3 (GBP1) are shown. The pivot point residues and the generated helix α4′ variants for GBP1 are marked. GBP human guanylate-binding protein, ATL human atlastin.
